# Multi-targeting therapeutic mechanisms of the Chinese herbal medicine QHD in the treatment of non-alcoholic fatty liver disease

**DOI:** 10.18632/oncotarget.15482

**Published:** 2017-02-18

**Authors:** Qin Feng, Wensheng Liu, Susan S. Baker, Hongshan Li, Cheng Chen, Qian Liu, Shijie Tang, Lingyu Guan, Maria Tsompana, Rafal Kozielski, Robert D. Baker, Jinghua Peng, Ping Liu, Ruixin Zhu, Yiyang Hu, Lixin Zhu

**Affiliations:** ^1^ Institute of Liver Disease, Shuguang Hospital, Shanghai University of Traditional Chinese Medicine, Shanghai, China; ^2^ Digestive Diseases and Nutrition Center, Women and Children's Hospital of Buffalo, Buffalo, NY, USA; ^3^ Department of Pediatrics, The State University of New York at Buffalo, Buffalo, New York, USA; ^4^ Ningbo No.2 Hospital, Ningbo, Zhejiang Province, China; ^5^ Department of Bioinformatics, Tongji University, Shanghai, China; ^6^ Center of Excellence in Bioinformatics and Life Sciences, The State University of New York at Buffalo, Buffalo, New York, USA; ^7^ Institute of Digestive Diseases, Longhua Hospital, Shanghai University of Traditional Chinese Medicine, Shanghai, China; ^8^ Women and Children's Hospital of Buffalo, Buffalo, NY, USA; ^9^ Department of Pathology, The State University of New York at Buffalo, Buffalo, New York, USA

**Keywords:** NAFLD, lipid synthesis, anti-oxidant, gut microbiome, Treg

## Abstract

Beneficial effects of the Chinese herbal medicine Qushi Huayu Decoction (QHD) were observed with non-alcoholic fatty liver disease (NAFLD) patients and animal models. The impact of QHD or its active components (geniposide and chlorogenic acid, GC) on NAFLD liver transcriptome and gut microbiota was examined with NAFLD rats. Increased expression for genes required for glutathione production and decreased expression for genes required for lipid synthesis was observed in NAFLD livers treated with QHD and GC. GC treatment decreased serum LPS, which could be explained by reduced mucosal damage in the colon of GC-treated rats. Further, our data suggest an increased abundance of Treg-inducing bacteria that stimulated the Treg activity in GC treated colon, which in turn down-regulated inflammatory signals, improved gut barrier function and consequently reduced hepatic exposure to microbial products. Our study suggests that QHD simultaneously enhanced the hepatic anti-oxidative mechanism, decreased hepatic lipid synthesis, and promoted the regulatory T cell inducing microbiota in the gut.

## INTRODUCTION

Non-alcoholic fatty liver disease (NAFLD) refers to a spectrum of conditions, including simple steatosis, non-alcoholic steatohepatitis (NASH), fibrosis and cirrhosis. As the hepatic component of the metabolic syndrome, NAFLD is characterized by hepatocellular lipid accumulation without excessive alcohol intake. NAFLD has become the most common chronic liver disease, along with increased prevalence of obesity and metabolic syndrome [1]. End-stage NAFLD is projected to be the leading cause for liver transplantation in US by 2020 [2]. According to the most current hypothesis, “multiple hits” contribute to the development of severe NAFLD or NASH [3], including, but not limited to oxidative stress as a consequence of excessive lipid oxidation, gut microbiome-related challenges, cytokines produced in the adipose tissue, innate immunity, endoplasmic reticulum (ER) stress and genetic predisposition.

Therapies targeting one component of NAFLD pathology have achieved limited results so far [4–6]. One explanation for their limitation is that different components of NAFLD are inter-convertible. For example, liver fat accumulation or steatosis can cause oxidative stress and consequently activate inflammatory reactions. In agreement, studies on the natural history of NAFLD have revealed that a sizable fraction of simple steatosis progresses to severe NAFLD stages including liver inflammation, fibrosis and cirrhosis [7]. Also, research has supported the opposite direction for the development of NAFLD, with inflammation leading to liver steatosis [8]. Therefore, novel strategies integrating multiple parallel interventions are desirable for treating this serious disease.

Traditional Chinese medicine has upheld the holistic therapeutic philosophy for more than 2000 years [9]. Many Chinese herbal formulae are multi-targeting in the treatment of NAFLD [4, 10]. In China one of the popular formulae for NAFLD treatment is the Qushi Huayu decoction (QHD), a mixture of five herbs including *Artemisia capillaries Thunb*, *Gardenia jasminoides Ellis*, *Fallopia japonica*, *Curcuma longa L*., and *Hypericum japonicum Thunb*. QHD was developed from the Yinchenhao decoction, a classical prescription documented in the book *Treatise on Cold Damage Disorders*, dating back to the Han dynasty (206 BC-220 AD), when the Yinchenhao decoction had become a popular and effective therapy for jaundice. In recent times, the hepatoprotective effect of the Yinchenhao decoction was further manifested in patients with infectious hepatitis [11, 12], alcoholic [13] and non-alcoholic fatty liver disease [14–16].

Our previous research showed that QHD treatment reduces serum ALT/AST, triglyceride (TG), cholesterol and liver fat (by ultrasound) [17]. Also, QHD therapy improved the Insulin Resistance-Homeostasis Model Assessment (IR-HOMA) index, although patients remained insulin-resistant [18]. Furthermore, we demonstrated that QHD therapy consistently achieved better outcomes on every disease marker than the hepatoprotective compound polyenylphosphatidylcholine and confirmed its beneficial effect in NAFLD animal models [14–16]. Despite the observed efficacy of QHD as a NAFLD treatment, knowledge on its therapeutic mechanisms remains limited. In NAFLD animal and cell culture models we showed that QHD activates AMP-activated protein kinase (AMPK) and contributes to liver fat reduction [14]. This finding is in line with the anti-steatotic effect of *A. capillaries*, a major component of QHD, which also exhibits antioxidant, anti-inflammatory, and antifibrotic activities in various disease models (reviewed at [19]). The antioxidant activity of *A. capillaries* is mainly attributed to chlorogenic acid, a major active ingredient found in the aqueous [20] and methanol [21] extracts of *A. capillaries*. Another major component of QHD, *Gardenia jasminoides Ellis*, exhibits anti-inflammatory activity attributed to its major active ingredient geniposide [22]. We have also observed that a recipe composed of geniposide and chlorogenic acid (GC) can significantly improve experimental fatty liver [23].

Herein, to better understand the therapeutic mechanisms of QHD, we examined the effects of QHD treatment on the liver transcriptome and gut microbiome of NAFLD rats and identified multiple therapeutic targets of QHD: genes responsible for glutathione (GSH) production, genes required for lipid synthesis and the regulatory T cell inducing microbiota in the gut.

## RESULTS

### Therapeutic effect of QHD and GC therapies on the HFD-fed rat model of NAFLD

**Figure 1:**
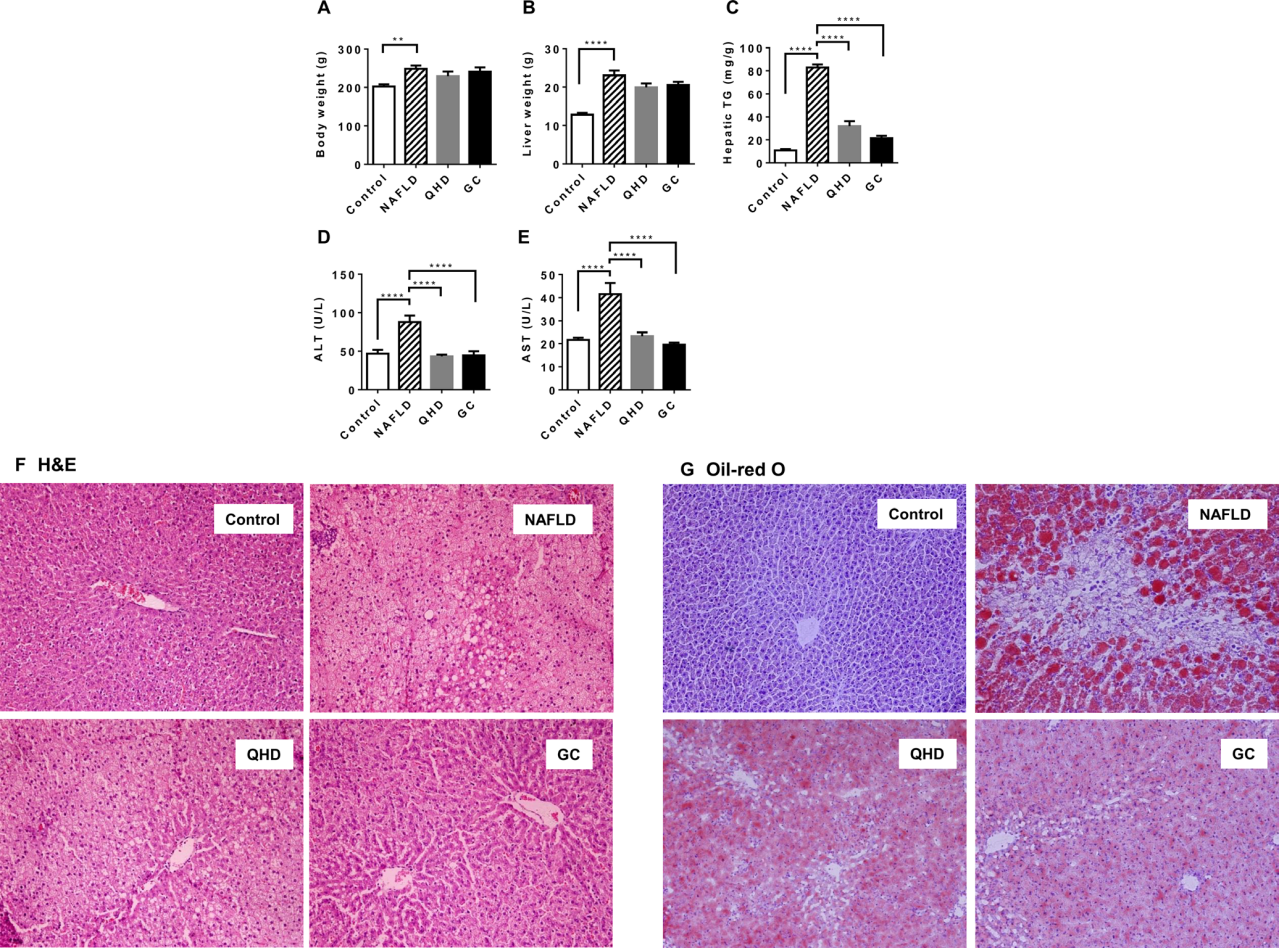
Beneficial effects of QHD and GC therapies on the fatty liver in the high-fat diet fed rat model Bar graphs are the plots of end-point body weight (**A**), liver weight (**B**), hepatic TG (**C**), serum ALT (**D**) and AST (**E**) of the study groups: (I) control rats (Control), (ii) NAFLD rats (NAFLD), (iii) NAFLD rats treated with Qushi Huayu Decoction (QHD), and (iv) NAFLD rats treated with GC (GC). Plotted values are mean ± SEM. *N* = 8 or 9 for all groups. *p < 0.05; **p < 0.01; ***p < 0.001, ****p < 0.0001, post-hoc Dunnett's test. Liver sections were stained with hematoxylin/eosin (**F**) and oil red O (**G**).

To investigate the molecular therapeutic mechanism of QHD, we conducted a liver gene expression study with a high fat diet (HFD) fed rat model. Global microarray gene expression analysis was performed with four groups of rats fed: (i) standard chow for 8 weeks (control group), (ii) HFD for 8 weeks (NAFLD group), (iii) HFD for 8 weeks with QHD therapy during the last 4 weeks (QHD group), and (iv) HFD for 8 weeks with GC therapy during the last 4 weeks (GC group). Compared to control rats, rats fed HFD (NAFLD rats) exhibited higher body weight, liver weight, liver TG, and serum ALT/AST, indicating metabolic disorders and NAFLD symptoms (Figure 1A–1E). Although the four-week long treatment with QHD or GC did not impact body (Figure 1A) and liver weight (Figure 1B) of NAFLD rats, both treatments decreased liver TG (Figure 1C), serum ALT (Figure 1D) and AST (Figure 1E) to similar levels as control rats. Pathological examination of liver sections revealed frequent incidence of macrosteatosis and hepatocyte ballooning in NAFLD rats, which was ameliorated in livers of QHD- and GC-treated rats (Figure 1F). Consistent with the biochemical analysis of liver TG, the Oil-red O staining revealed increased fat deposition in the liver of NAFLD rats, and that QHD and GC treatments reduced liver fat content (Figure 1G).

### Genes and pathways associated with NAFLD pathology

**Figure 2:**
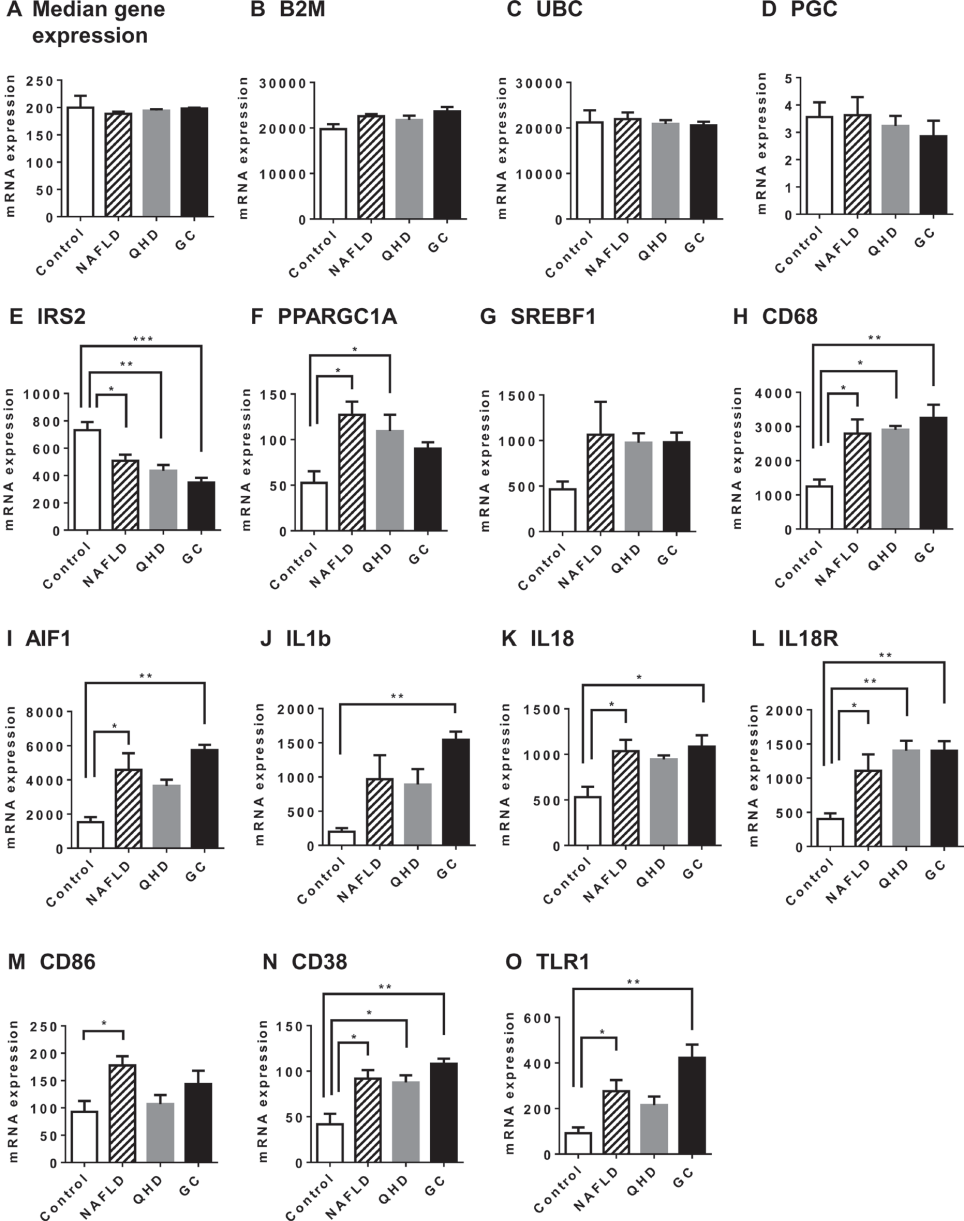
Altered gene expression in NAFLD rat model: genes associated with NAFLD pathology Global gene expression was examined by microarray analysis. Median gene expression (**A**) was similar among all study groups. Reference genes B2M (**B**), UBC (**C**) and PGC (**D**) exhibited similar expression among all study groups. IRS2 (**E**) is required for insulin signaling; PPARGC1A (**F**) and SREBF1 (**G**) regulate lipid and carbohydrate metabolism; CD68 (**H**) is a surface marker for Kupffer cells; AIF1(**I**) is a marker for macrophage; IL1b (**J**), IL18 (**K**) and IL18R (**L**) are mediators of inflammatory response; CD86 (**M**) mediates T cell activation; CD38 (**N**) is a marker for activated stellate cells; TLR1(**O**) is a receptor for peptidoglycan and lipoprotein. Plotted values are the mean ± SEM of the mRNA expression levels in the livers of control rats (Control), NAFLD rats (NAFLD), NAFLD rats treated with Qushi Huayu Decoction (QHD), and NAFLD rats treated with GC (GC). *N* = 3 for all groups. *p < 0.05; **p < 0.01; ***p < 0.001, ****p < 0.0001, post-hoc Dunnett's test.

Expression levels of ~40,000 transcripts were examined using rat livers of the study groups on a global microarray platform. Most transcripts were similarly expressed among all examined groups. Similar median gene expression and expression levels for reference genes (Figure 2A–2D) are indicative of similar experimental conditions and quality of data. A total of 1150 transcripts were differentially expressed between the NAFLD and the control group. Enriched pathways with NAFLD upregulated genes include inflammatory signaling, diabetes mellitus signaling, and pattern recognition receptor mediated signaling, expected from NAFLD livers (Supplementary Table 1). We identified relatively few enriched pathways with NAFLD downregulated genes (Supplementary Table 2).

As to individual genes (Figure 2), NAFLD rats exhibited decreased expression of IRS2 when compared to control animals, reflective of impaired insulin signaling in NAFLD livers. Elevated activities of lipid and carbohydrate metabolism are characteristics of NAFLD livers. Consistently, increased expression of PPARγ and a trend of increased expression for SREBF1 were observed in NAFLD rat livers. Elevated expression of CD68 (Kupffer cell marker), AIF1 (macrophage marker), IL1b, IL18, IL18R, and CD86 (mediator of T cell activation) were also observed in NAFLD rat livers indicating liver inflammation. Increased expression of CD38 (marker for activated stellate cell) and TLR1 (receptor for peptidoglycan and lipoprotein) in NAFLD rats was also consistent with a successful NAFLD model. However, no significant difference was observed between NAFLD and treatment groups for genes selected in Figure 2.

### Genes and pathways mediating the therapeutic effects of QHD/GC treatment

**Figure 3:**
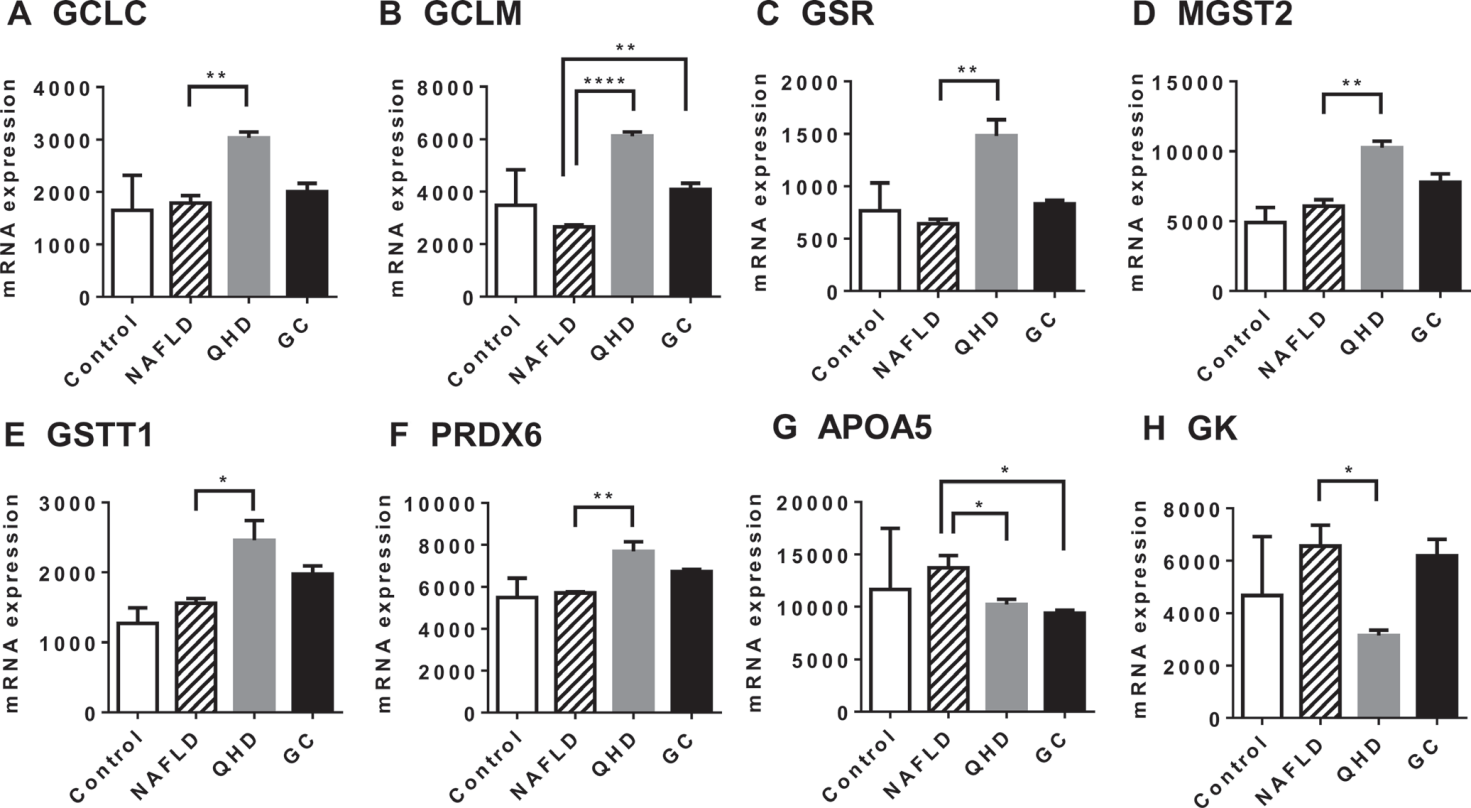
Altered gene expression upon herbal treatment in NAFLD rat model: genes associated with improved liver health Global gene expression was examined by microarray analysis. GCLC (**A**) and GCLM (**B**) are required in the de novo synthesis of glutathione; GSR (**C**) catalyzes the reduction of glutathione disulfide (GSSG); MGST2 (**D**) catalyzes the conjugation of leukotriene A4 and reduced glutathione to produce leukotriene C4; GSTT1 (**E**) catalyzes the conjugation of reduced glutathione to a variety of electrophilic and hydrophobic compounds; PRDX6 (**F**) encodes an enzyme that catalyzes the reduction of peroxide by ascorbic acid or glutathione; APOA5 (**G**) is a marker for steatosis; GK (**H**) is required in triglyceride synthesis. Plotted values are the mean ± SEM of the mRNA expression levels in the livers of control rats (Control), NAFLD rats (NAFLD), NAFLD rats treated with Qushi Huayu Decoction (QHD), and NAFLD rats treated with GC (GC). *N* = 3 for all groups. *p < 0.05; **p < 0.01; ***p < 0.001, ****p < 0.0001, post-hoc Dunnett's test.

To identify genes and pathways associated with QHD/GC therapy, pathway enrichment analysis was performed with the differentially expressed genes (DEGs) between the NAFLD and treatment groups. QHD therapy had profound impact on gene expression in the livers of NAFLD rats. Compared to the NAFLD group, elevated expression of 1620 transcripts and decreased expression of 1432 transcripts was observed in the livers of QHD-treated rats. Pathway analysis performed with upregulated and downregulated genes in the QHD-treated group unearthed a number of enriched pathways, among which the xenobiotic metabolism signaling pathway was elevated in the QHD group (Supplementary Table 3). This is further evidence of the quality of our microarray dataset, since the herbal mixture of QHD is a rich source of xenobiotic substances. It is outstanding that among the elevated pathways several are related to the antioxidant glutathione, such as glutathione-mediated detoxification, glutathione redox reactions I, glutathione biosynthesis, aryl hydrocarbon receptor signaling, xenobiotic metabolism signaling, NRF2-mediated oxidative stress response, and γ-glutamyl cycle. Elevated expression of key enzymes involved in glutathione biology are shown in Figure 3, including GCLC, GCLM, GSR, MGST2, GSTT1 and PRDX6. QHD treatment also caused downregulation of important pathways, such as cysteine degradation (Supplementary Table 4) that would allow higher availability of cysteine, a substrate for glutathione synthesis.

Consistent with reduced liver fat, QHD-treated livers exhibited reduced expression of APOA5 (Figure 3G), an indication of reduced lipid droplet in liver. However, enrichment analysis did not identify any major pathways that directly impact liver fat. Examination of enzymes in the pathways of fatty acid uptake, fatty acid *de novo* synthesis, fatty acid β-oxidation and VLDL secretion (major routes to add or remove fat in liver [24]) revealed no difference between NAFLD and QHD groups. Instead, the enzyme required for the synthesis of triglyceride, glycerol kinase, exhibited a 50% decrease in QHD-treated livers compared to NAFLD livers (Figure 3H).

Similarly, GC treatment had a large impact on NAFLD liver gene expression. Compared to the NAFLD group, elevated expression of 1372 transcripts and decreased expression of 908 transcripts was observed in the livers of GC-treated rats. Pathway analysis using the list of upregulated or downregulated genes identified a number of enriched pathways (Supplementary Tables 5 and 6). Like in QHD-treated livers, genes and pathways involving the antioxidant glutathione were elevated in GC-treated livers, including genes GSR, GSTT1 and PRDX6 and the pathway Glutathione Redox Reactions I (Supplementary Table 5).

**Figure 4:**
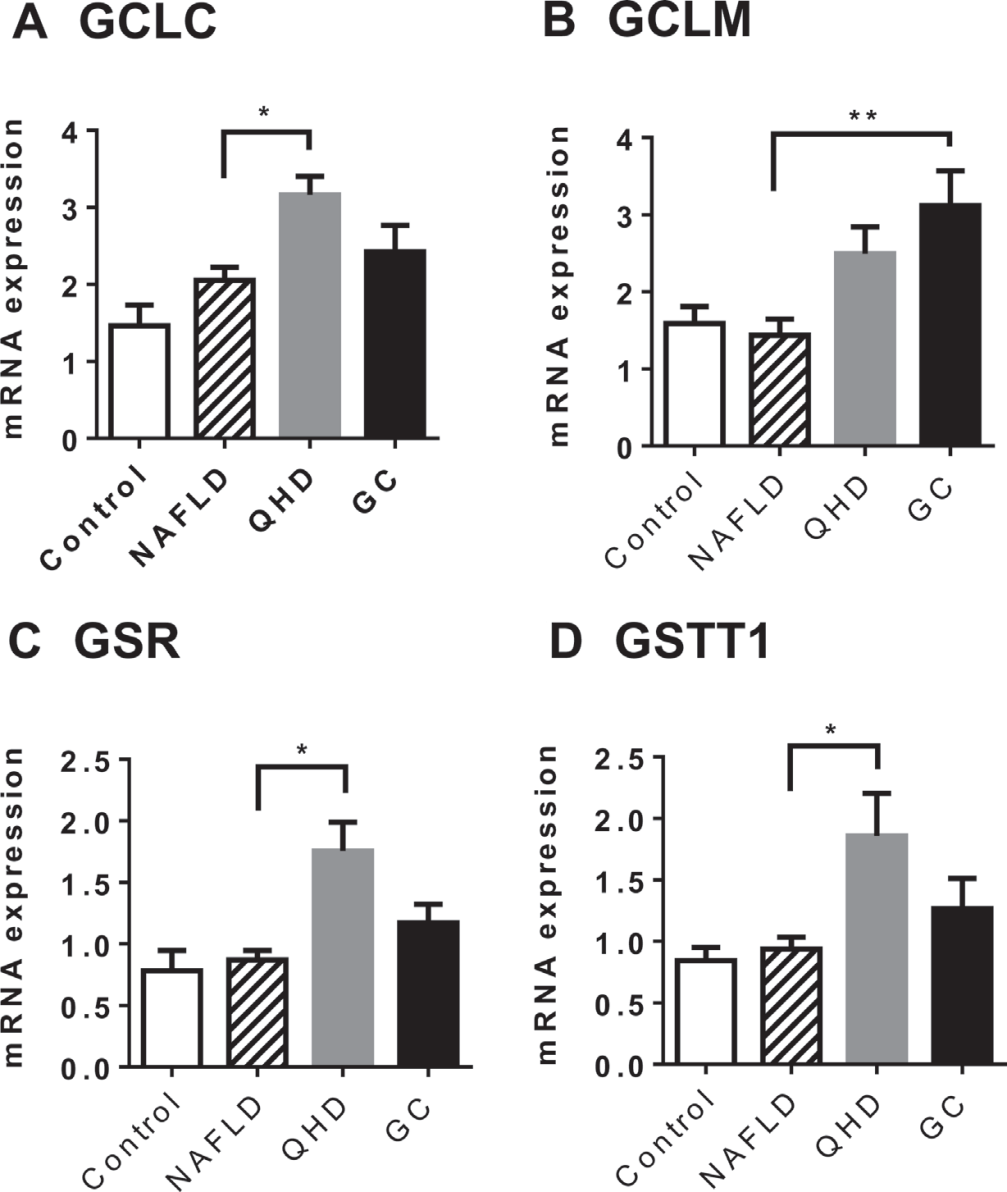
Altered gene expression upon herbal treatment in NAFLD rat model: genes associated with improved liver health Gene expression was examined by qRT-PCR. GCLC (**A**) and GCLM (**B**) are required in the de novo synthesis of glutathione; GSR (**C**) catalyzes the reduction of glutathione disulfide (GSSG); GSTT1 (**D**) catalyzes the conjugation of reduced glutathione to a variety of electrophilic and hydrophobic compounds. Plotted values are the mean ± SEM of the mRNA expression levels in the livers of control rats (Control), NAFLD rats (NAFLD), NAFLD rats treated with Qushi Huayu Decoction (QHD), and NAFLD rats treated with GC (GC). *N* = 6 for all groups. *p < 0.05; **p < 0.01; post-hoc Dunnett's test.

**Figure 5:**
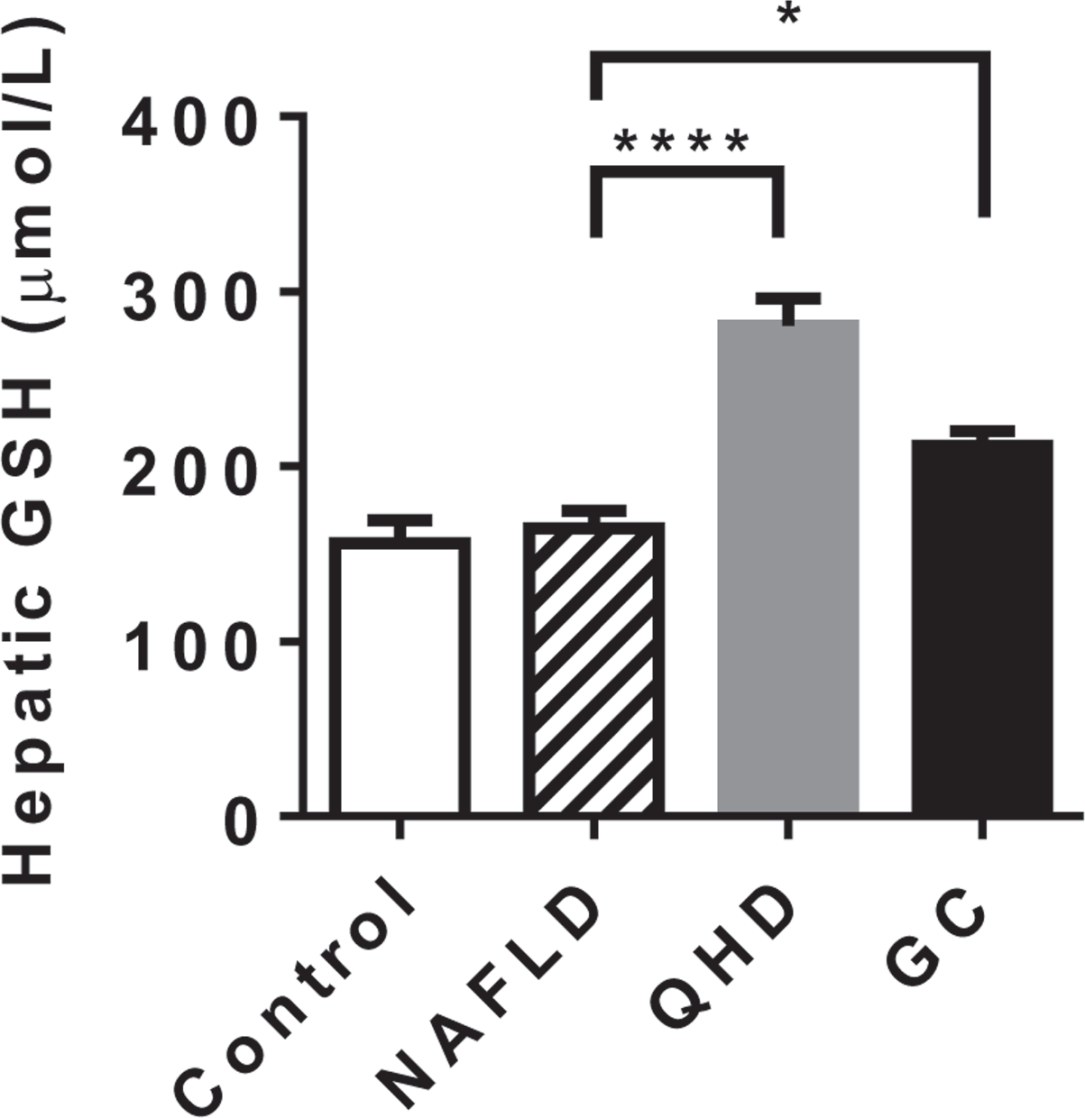
Treatment with QHD and GC increased hepatic glutathione levels *N* = 6 for all groups. *p < 0.05; ****p < 0.0001, post-hoc Dunnett's test.

To validate the microarray analysis data, some of the GSH metabolism related genes were analyzed in quantitative real-time PCR (qRT-PCR) and showed similar results (Figure 4). These results prompted us to examine the hepatic GSH levels of the study groups. Similar GSH levels were observed between control and NAFLD groups, while QHD and GC groups exhibited elevated GSH levels compared to the NAFLD group (Figure 5).

### Impact of GC therapy on NAFLD gut microbiome

**Figure 6:**
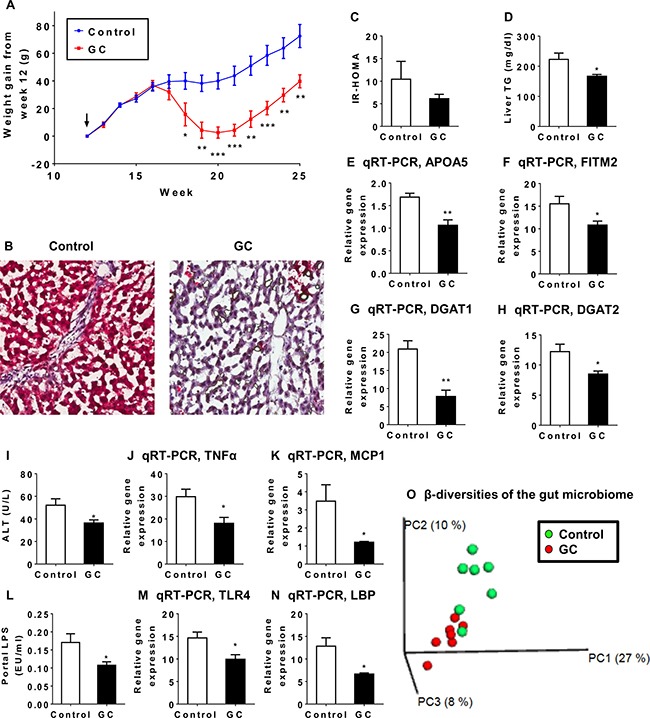
The therapeutic effect of GC treatment is associated with alteration in the gut microbiome The NAFLD rat model was established with high-fat diet plus DSS treatment for 12 weeks. NAFLD rats were then divided into a control group (*N* = 7) and a GC treatment group (*N* = 7). After 13 weeks of GC treatment, animals were sacrificed to evaluate blood biochemistry, liver pathology and liver gene expression. (**A**) Rats exhibited decreased weight gain 6 weeks after GC treatment. Arrow indicates the start of GC treatment. Plotted values are mean ± SEM. Statistical significance for the difference between GC and control groups is indicated: *p < 0.05; **p < 0.01; ***p < 0.001. (**B**) Oil-red O staining revealed reduced fat accumulation and inflammatory foci in the liver of GC-treated rats. Original amplification: 200X. (**C**) Insulin resistance (IR-HOMA) did not differ between GC and control groups. (**D**) GC treatment reduced liver triglyceride (TG) content. Reduced mRNA expression was observed for APOA5 (**E**) and FITM2 (**F**), both encoding key proteins for lipid droplet biology. Reduced mRNA expression was also observed for genes required for TG synthesis (**G**, DGAT1; **H**, DGAT2). GC-treated rats exhibited decreased liver ALT (**I**) and decreased mRNA expression for proinflammatory cytokine TNF α (**J**) and chemokine MCP1 (**K**). GC-treated rats exhibited decreased serum level of LPS (**L**) and decreased hepatic expression of LPS receptors TLR4 (**M**) and LBP (**N**). (**O**) Weighted UniFrac-based principle coordinates analysis revealed that most of the gut microbiome samples clustered by type of treatment, indicating a distinct population structure for GC-treated samples versus control.

To investigate whether QHD therapy has an impact on the gut microbiome, a rat model of HFD plus dextran sulfate sodium (DSS) feeding was employed [25]. As the active components geniposide and chlorogenic acid seem to mediate the major therapeutic effects of QHD therapy, the chemically defined GC therapy was used in the microbiome study. Rats were fed HFD and DSS for 12 weeks to establish a NAFLD model. These rats exhibited higher body weight, liver triglycerides, and serum ALT compared to rats fed standard chow (data not shown). While these rats were still on HFD plus DSS feeding, they were divided into two groups: a GC therapy and a control group. GC therapy was then conducted for 13 weeks before rats were sacrificed to evaluate the effects of GC. GC therapy reduced weight gain after 5 weeks of GC treatment (Figure 6A). The effect of GC on weight gain persisted thereafter (Figure 6A). Importantly, Oil-red O staining demonstrated that GC therapy reduced liver fat and inflammation (Figure 6B), although insulin resistance was not altered (Figure 6C). Consistently, decreased liver TG (Figure 6D) and gene expression for lipid droplet proteins ApoA5 (Figure 6E) and FITM2 (Figure 6F) were observed in GC-treated rats. Reduced expression in genes required for TG synthesis, DGAT1 (Figure 6G) and DGAT2 (Figure 6H), provided an explanation for reduced liver fat. Reduced serum ALT (Figure 6I), expression of inflammatory cytokine TNFα (Figure 6J) and chemokine MCP1 (Figure 6K) were indicative of ameliorated liver injury and supported a reduced liver inflammation in GC-treated rats. Reduced serum lipopolysaccharides (LPS, Figure 6L) suggested that reduced hepatic exposure to gut microbial products contributed to the beneficial effect of GC on liver inflammation. This was confirmed by reduced gene expression of LPS receptors TLR4 (Figure 6M) and LBP (Figure 6N) in GC-treated livers.

**Figure 7:**
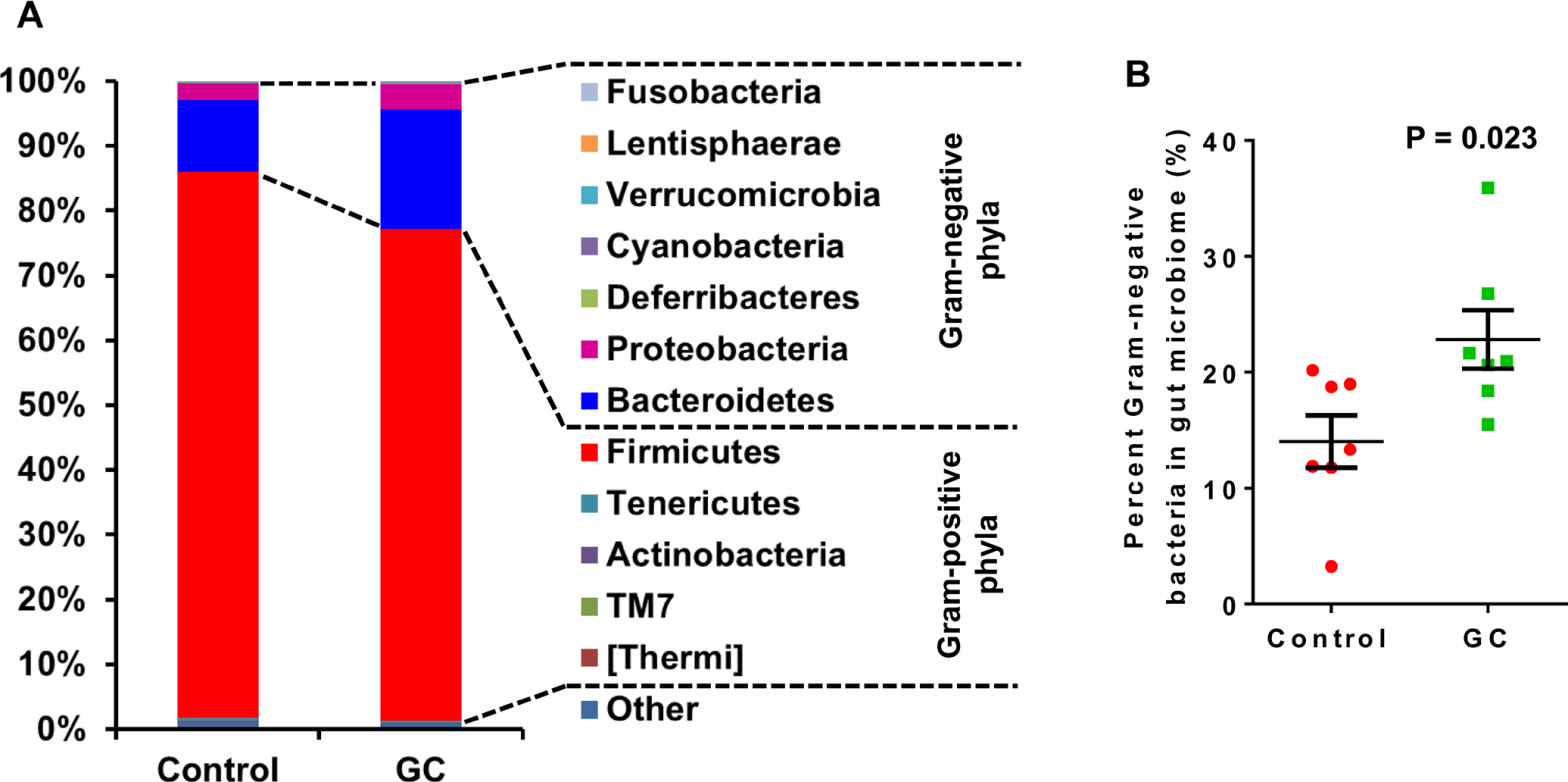
Altered gut microbial composition at phylum level in GC-treated rats Control rats were NAFLD rats induced with high fat diet and DSS; GC rats were NAFLD rats treated with GC for 13 weeks. (**A**) Average phylum distribution of the gut microbiome of control and GC groups. Out of 12 phyla observed in the gut of the rats, Fusobacteria, Lentisphaerae, Verrucomicrobia, Cyanobacteria, Deferribacteres, Proteobacteria and Bacteroidetes are Gram-negative bacteria; Firmicutes, Tenericutes, Actinobacteria, TM7, and Thermi are Gram-positive bacteria. Sequences not assigned to any known phylum (Other) comprise a small fraction of the microbiome in both groups (Control: 1.01%; GC: 0.76%). (**B**) Percent Gram-negative bacteria in gut microbiome. Error bars are standard errors of the means. *P*-values are indicated.

One possible cause for reduced serum LPS is decreased Gram-negative bacteria in the gut. To test this hypothesis, colonic contents were analyzed for microbiome composition by 16S rRNA sequencing. Weighted UniFrac analysis of β-diversities showed a clear trend of separation of the GC-treated gut microbiota from the microbiota of control animals (Figure 6O). This was strong evidence that GC therapy had a large impact on the structure of the gut microbiome. Indeed, differences at every taxonomic level were observed between GC-treated rats and controls (Table 1). A total of 12 bacterial phyla were identified from all samples. Among these Bacteroidetes [26], Cyanobacteria [27], Deferribacteres (all sequences are Gram-negative genus Mucispirillum [28]), Fusobacteria [29], Lentisphaerae [30], Proteobacteria [26] and Verrucomicrobia [31] are Gram-negative; Actinobacteria [26], Firmicutes [26], TM7 [32], Tenericutes [31] and Thermi [33] are Gram-positive. Small fractions of the 16S rRNA sequences were not assigned to any taxon (Control: 1.01%; GC: 0.76%). Against our expectation, GC treatment caused a significant increase in the abundance of Gram-negative bacteria in the gut (Figure 7), suggesting that the compositional change in the microbiota is not the cause of decreased serum LPS.

**Table 1:** Abundant taxa in the gut microbiome of the rats treated with GC and the control rats^1^

Description			Control^2^			Treatment^3^			Fold change^4^			*P* value^5^		
p__Actinobacteria			0.30%			0.20%			0.68			0.371		
p__Bacteroidetes			11.15%			18.52%			1.66			0.020		
	f__Bacteroidaceae			5.76%			13.81%			2.40			0.011	
		g__Bacteroides			5.76%			13.81%			2.40			0.011
	f__Porphyromonadaceae			0.54%			0.59%			1.10			0.836	
		g__Parabacteroides			0.54%			0.59%			1.10			0.835
	f__Rikenellaceae			0.98%			1.18%			1.20			0.744	
		g__unknown6			0.89%			1.17%			1.31			0.643
	f__S24-7			2.46%			1.05%			0.43			0.008	
		g__unknown			2.46%			1.05%			0.43			0.008
	f__[Odoribacteraceae]7			0.48%			0.53%			1.09			0.746	
		g__Butyricimonas			0.38%			0.49%			1.31			0.325
	o__Bacteroidales;f__unknown6			0.78%			1.08%			1.39			0.155	
		g__unknown			0.78%			1.08%			1.39			0.155
p__Firmicutes			84.20%			75.96%			0.90			0.031		
	f__Enterococcaceae			0.34%			0.25%			0.72			0.642	
		g__Enterococcus			0.34%			0.25%			0.72			0.636
	f__Lactobacillaceae			0.43%			0.24%			0.57			0.286	
		g__Lactobacillus			0.43%			0.24%			0.57			0.286
	o__clostridiales;f__unknown			21.62%			6.86%			0.32			0.001	
		g__unknown			21.62%			6.86%			0.32			0.001
	f__Clostridiaceae			30.17%			52.19%			1.73			0.017	
		g__unknown			8.81%			24.91%			2.83			0.001
		g__Clostridium			0.09%			0.56%			6.56			0.002
		g__SMB53			21.10%			26.42%			1.25			0.304
	f__Lachnospiraceae			9.85%			3.92%			0.40			0.008	
		g__unknown			4.48%			1.47%			0.33			0.031
		g__[Ruminococcus]			0.45%			0.07%			0.15			0.005
		g__Coprococcus			0.90%			0.26%			0.29			0.007
		g__Dorea			3.50%			2.02%			0.58			0.181
	f__Peptococcaceae			1.75%			0.60%			0.34			0.129	
		g__rc4-4			1.35%			0.56%			0.41			0.266
	f__Peptostreptococcaceae			0.14%			0.40%			2.84			0.002	
		g__unknown			0.13%			0.35%			2.63			0.003
	f__Ruminococcaceae			18.29%			9.67%			0.53			0.071	
		g__unknwon			5.28%			3.35%			0.63			0.098
		g__Oscillospira			6.97%			2.46%			0.35			0.018
		g__Ruminococcus			5.96%			3.83%			0.64			0.464
	f__Erysipelotrichaceae			0.27%			0.40%			1.48			0.535	
		g__Allobaculum			0.01%			0.30%			39.76			0.121
p__Proteobacteria			2.49%			3.88%			1.56			0.217		
	o__RF32;f__unknown			1.05%			1.23%			1.17			0.748	
		g__unknown			1.05%			1.23%			1.17			0.748
	f__Alcaligenaceae			0.05%			1.11%			21.06			0.007	
		g__Sutterella			0.05%			1.11%			21.06			0.007
	f__Desulfovibrionaceae			1.19%			0.48%			0.40			0.054	
		g__unknown			0.57%			0.25%			0.44			0.146
	f__Enterobacteriaceae			0.02%			1.03%			66.13			< 0.001	
		g__unknown			0.02%			1.02%			67.27			< 0.001
p__Tenericutes			0.47%			0.25%			0.53			0.383		

1 Included in this table are taxa (phyla, families and genera) with average abundance greater than 0.3% in any of the study groups.

2 Average abundance in gut microflora of rats treated with water, *n* = 7.

3 Average abundance in gut microflora of rats treated with GC, *n* = 8.

4 GC/Control.

5 Two tailed Student t test or Mann Whitney test for normally distributed or not-normally distributed data.

6 Theoretical family or genus based on 16S rRNA sequence similarity clustering.

7 Names in brackets are not standard taxonomy, but are names proposed by greengenes curators.

**Figure 8:**
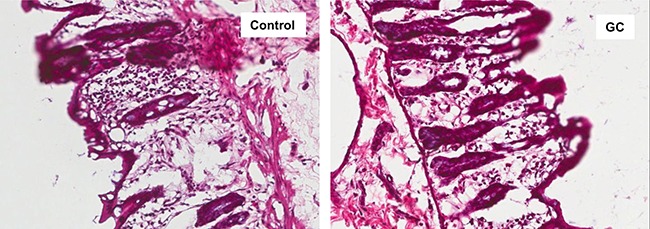
GC treatment ameliorated colon mucosal damage in NAFLD rats Hematoxylin and eosin staining of colon sections from control and GC groups are shown. Lymphocyte infiltration and colon mucosal damage were apparent in control rats, and less frequently observed in GC rats. Original amplification: 200X.

**Figure 9:**
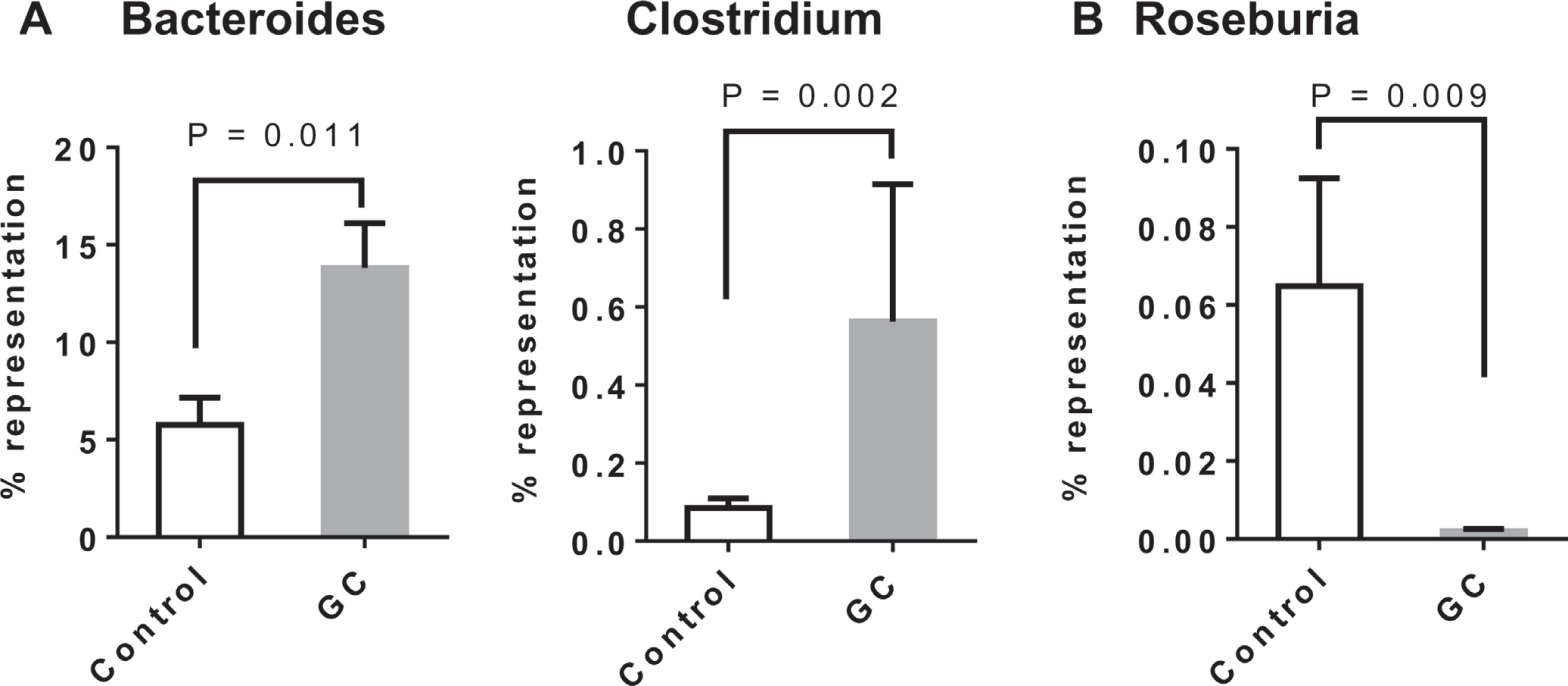
Altered gut microbial composition in GC-treated rats: increase of Treg-inducing and reduction of SCFA-producing bacteria (**A**) Alterations of genera Bacteroides and Clostridium in the gut of GC-treated rats. Several known Treg-inducing bacteria belong to these two genera. (**B**) Alterations of genus Roseburia in the gut of GC-treated rats. Other significant SCFA producing genera such as Faecalibacterium, Eubacterium and Prevotella were absent from most samples. Error bars are standard errors of the means. *P*-values are indicated.

Examination of colon histology revealed that GC treatment ameliorated colonic injury and incidence of inflammatory cell infiltration (Figure 8). There are two potential mechanisms for gut microbiota to regulate the inflammatory processes in the gut. Some bacterial species in the genera of Bacteroides [34, 35] and Clostridium [36, 37] can induce regulatory T cells (Treg) in the colon. Suppression of the inflammation by Treg cells could explain the improved gut barrier function in GC-treated rats. The other mechanism can involve bacterial fermentation products, short chain fatty acids (SCFA), which may strengthen the gut barrier function through glucagon-like peptide 2 mediated intestinotrophic effect, or through GPR43 mediated suppression of colitis [38]. Genera Faecalibacterium, Eubacterium, Roseburia [39, 40], and Prevotella [41] are known for their production of SCFA. Examination of the microbial composition revealed that GC treatment caused elevated representation of both Bacteroides and Clostridium in the gut (Figure 9A, 9B). On the other hand, Roseburia was decreased in the gut of the GC group (Figure 9C) while other known SCFA genera were barely detected.

**Figure 10:**
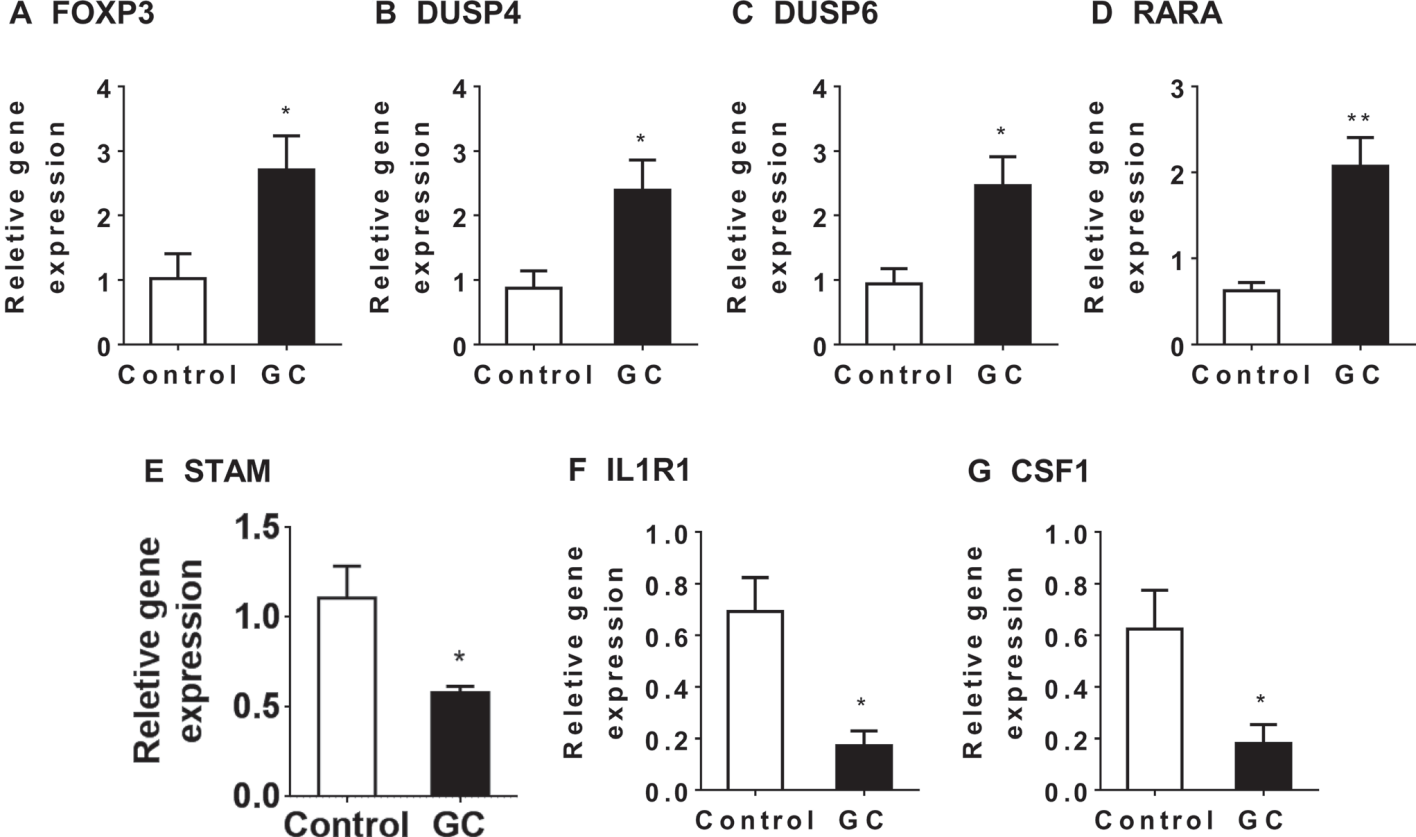
The impact of GC treatment on Treg-related genes in the colon of NAFLD mice Relative gene expression was determined for FOXP3 (**A**), DUSP4 (**B**), DUSP6 (**C**), RARA (**D**), STAM (**E**), IL1R1 (**F**) and CSF1 (**G**) in the colon of NAFLD mice treated with GC (GC, *N* = 8) or water (control, *N* = 6). Plotted values are mean ± SEM. Statistical significance for the difference between GC and control groups is indicated: *p < 0.05; **p < 0.01.

Thus microbiome analysis supported altered Treg activity in the GC-treated colon. Consequently, we examined mRNA expression of Treg specific genes in the colon of NAFLD mice by qRT-PCR. The expression of FOXP3 in GC-treated colons was 2.7 fold that of the controls (*P* = 0.033, Figure 10A). Similarly, expression of other Treg-associated genes such as DUSP4, DUSP6, and RARA [42] were elevated in GC-treated colons (Figure 10B–10D). Consistently, genes required for inflammatory signaling, such as STAM, IL1R1 and CSF1, were decreased in GC-treated colons. (Figure 10E–10G)

## DISCUSSION

**Figure 11:**
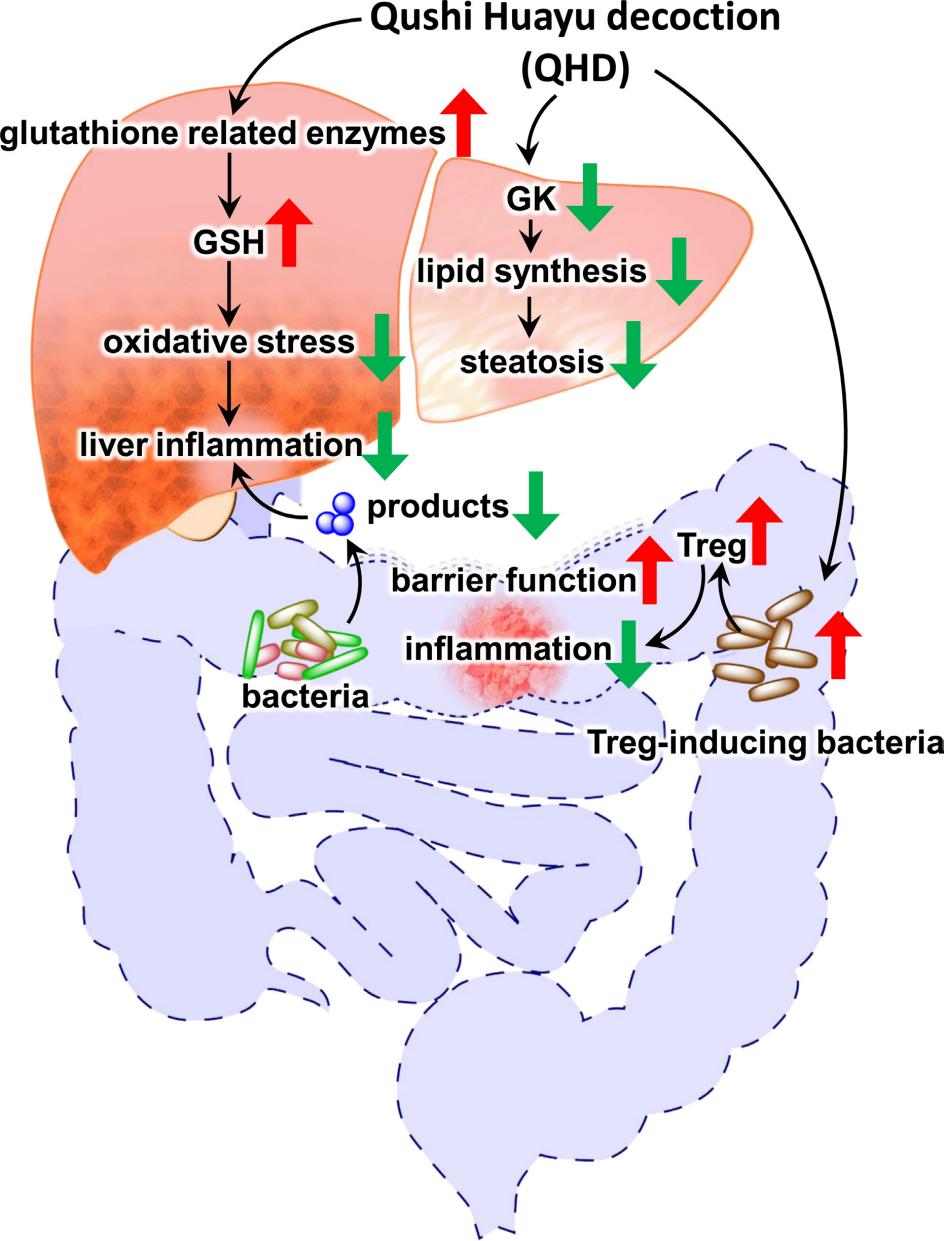
Summary of the multi-targeting therapeutic mechanisms of Qushi Huayu Decoction (QHD) for NASH Data presented in this report supports three independent mechanisms: (i) QHD induces GSH producing enzymes in the liver, leading to reduced oxidative stress and liver inflammation; (ii) QHD suppresses hepatic GK expression, causing reduced lipid synthesis and improving steatosis, (iii) QHD induces colonic Treg-inducing bacteria, leading to elevated Treg activity, and consequently decreased colonic inflammation, which in turn improves gut barrier function, decreases hepatic exposure to gut microbial products, and eventually leads to reduced liver inflammation. Red arrows indicate increased expression or activity; green arrows indicate decreased expression or activity.

We observed large and broad effects of the Chinese herbal medicine QHD, on the liver gene expression and the gut microbiome composition in NAFLD rats. Many of the changes in liver gene expression reflect decreased liver fat content, inflammation and injury. These observations along with elevated xenobiotic metabolism (herbal medicine is a rich source of xenobiotic substance) provide an assurance of the quality of the microarray datasets, from which, two mechanisms that may mediate the beneficial effect of QHD were identified: (i) elevated antioxidant capacities, and (ii) reduced TG synthesis. Similar hepatic and systemic beneficial effects were observed when NAFLD rats were treated with a combination of geniposide and chlorogenic acid (GC), two major active components of QHD. In addition, GC treatment boosted the bacterial genera to which Treg-inducing species belong, which is in line with increased Treg markers and reduced inflammatory markers in the colonic mucosa, improved gut barrier function, reduced LPS in the portal vein, reduced hepatic exposure to LPS and other gut microbial products, and eventually improved NAFLD symptoms. These observations support a multi-targeting mechanism for QHD in the treatment of NAFLD (Figure 11).

### Increased production of glutathione and usage of glutathione in antioxidant reactions

Out of the 40 pathways that exhibited elevated activities upon QHD treatment compared to the NAFLD group, several were related to the production of glutathione or the use of glutathione as an antioxidant. Elevated oxidation of fatty acids [43] and endogenous alcohol [44] contribute to excessive reactive oxygen species (ROS) in the NAFLD liver. Elevated ROS levels are reflected by induction of several antioxidant enzymes such as catalase, hemoglobin and paraoxonase 1 [45–47]. Apparently, the total anti-oxidative capacity is insufficient to contain the ROS in NAFLD livers. Elevated levels of peroxidation products exist in NAFLD livers [48] and blood [49]. Oxidative stress may cause liver injury and inflammation and is considered a major “hit” in the pathogenesis of NAFLD [3]. Therefore, an intervention targeting the oxidative status of the liver is desirable for NAFLD management, and QHD seems to be offering this advantage. Our data showed that QHD treatment boosted the expression levels of enzymes responsible for production of glutathione and for use of glutathione in antioxidant reactions. In line with this, reduced activity of cysteine degradation pathways allows more substrate cysteine for glutathione synthesis. We also observed an increased concentration of glutathione in rat NAFLD livers upon QHD treatment further supporting an increased antioxidant capacity in the QHD-treated NAFLD liver. These observations, at least in part, provide an explanation for reduced liver injury and inflammatory markers in QHD-treated rats.

To better appreciate the beneficial effect of QHD, it is worth noting that QHD treatment upregulated the expression of GCLC, which encodes the rate-limiting enzyme for GSH production. Previous studies have identified an association between NAFLD and the polymorphism in the GCLC promoter region [50]. Further, GCLC gene-knockout caused GSH depletion, hepatic inflammation, steatosis and liver failure [51]. These observations indicate that GCLC is a potential target for NAFLD intervention and likely a major mediator of the therapeutic effect of QHD.

One interesting link between the current study and our previous work is that QHD selectively boosted glutathione-related pathways, while its impact on other antioxidant enzymes were not impressive. In the liver of NAFLD patients, peroxidases catalase, hemoglobin and paraoxonase 1 are induced, [45–47] but the expression levels of GPX1 and GSR genes are comparable to those of normal livers, [47], suggesting a normal activity in glutathione metabolism in the NAFLD liver. This makes glutathione a better target for intervention, as other antioxidative mechanisms are already elevated and thus less likely to increase further.

### Reduced TG synthesis

Increased liver fat is the first “hit” in the pathogenesis of NAFLD and its removal is a desired intervention for NAFLD. QHD and GC therapy consistently removed liver fat from NAFLD patients and animal models. Our previous work suggests one mechanism whereby QHD treatment activates AMPK, and consequently inhibits de novo fatty acid synthesis [14]. Using our transcriptome data herein we aimed to identify the potential mechanism for reduced liver fat at the transcription level.

Major pathways that add or remove fat from hepatocytes include fatty acid uptake, fatty acid de novo synthesis, fatty acid oxidation and VLDL secretion [24]. Examination of our microarray data revealed no change in the transcription activities of the relevant genes. However, there was decreased expression of GK in QHD-treated livers, which predicts decreased TG production. Unexpectedly, the GK expression was not altered in GC-treated livers. To further complicate the issue, two other genes required for TG synthesis, DGAT1 and DGAT2, were downregulated by GC in rat NAFLD livers. Integrating these data, it is reasonable to conclude that QHD decreases hepatic TG synthesis through mRNA expression regulation of relevant genes, but the exact mechanism differs from that of GC therapy. To further test this hypothesis, future work is needed to identify the destination of fatty acids when TG synthesis is blocked in livers treated with QHD and GC.

### Altered gut microbiome and improved gut barrier function

Reduced serum LPS was observed in rats treated with GC, the major active components of QHD. This is consistent with decreased hepatic expression of TLR4 and LBP. These observations are of important clinical significance as many NAFLD patients exhibit elevated serum LPS levels [52]. Studies with patients and animal models have suggested that TLR4-mediated signaling is a potent driving force for the disease progression of NAFLD [53, 54]. In support of the role of gut-derived endotoxin in the pathogenesis of NAFLD, increased prevalence of NAFLD was observed in patients with inflammatory bowel diseases [55, 56].

The reduced serum LPS level indicated that gut microbiota is involved in the therapeutic mechanism of GC and QHD. Examination of the gut microbiome revealed alterations in many bacterial taxa upon GC treatment. However, in contrast to reduced serum LPS, the percentage of Gram-negative bacteria was increased upon GC treatment. Reduced serum LPS was indeed a result of improved gut barrier function, or ameliorated colon inflammation in GC-treated rats.

Gut microbiota may influence the gut barrier function via several mechanisms. Firstly, commensal bacteria are part of the gut barrier function, competing with pathogenic bacteria for resources and space. This mechanism seems to be irrelevant in QHD or GC therapy as the gut microbiota in NAFLD patients and animal models had abundant commensal bacteria before QHD and GC treatment. Secondly, several bacterial species are known for higher production of SCFA [39–41]. SCFAs are intestinotrophic, induce Treg cells and suppress colon inflammation (reviewed at [38]) and thus are warriors of gut barrier function. However, the GC-treated microbiome exhibited reduced SCFA producing bacteria. Therefore, SCFAs are not likely mediating the therapeutic effect of GC. Finally, several members of the gut community are inducers of Treg cells. The Treg-inducing bacteria were elevated upon GC treatment. Increased Treg activity will in turn suppress colon inflammation and improve gut barrier function. This is in line with the reduced neutrophil infiltration in the colonic mucosa of the GC-treated animals.

The impact of GC treatment on the gut microbiome likely differs from that of QHD treatment. We previously observed that QHD treatment decreased the opportunistic pathogen Escherichia/Shigella and increased the SCFA producer Collinsella [15]. It seems that QHD treatment may improve the gut barrier function through other mechanisms in addition to the Treg mediated pathway identified in this study. Future studies comparing the differences between QHD and GC treatments on hepatic gene expression and gut microbiome may reveal further insights on the molecular therapeutic mechanisms of QHD treatment.

In summary, the data presented here highlights three independent mechanisms that may mediate the beneficial effects of QHD in NAFLD animal models: (i) QHD induces production of glutathione to counteract the damage of oxidative stress, (ii) decreased TG synthesis may explain the reduced liver fat in QHD-treated livers, and (iii) mediated by the gut microbiota, the active components of QHD induce Treg cells that improve the gut barrier function and lead to reduced hepatic exposure to LPS and other products of the gut microbiota. These mechanisms target different components of the NAFLD pathology. This multi-targeting QHD therapy may be a good match for the multi-hit driven NAFLD pathogenesis. Our study contributes to the understanding of the therapeutic mechanisms of QHD and lays the foundation for the invention of a chemically-defined therapy from natural products for treating NAFLD.

## MATERIALS AND METHODS

### Study design

Estimation of required sample size was conducted with the G*Power version 3.1.9.2. In microarray gene expression analysis, only genes exhibiting >2-fold change among study groups were considered to identify the most important genes/pathways affected by QHD/GC treatment. In one-way ANOVA, we used an effect size F = 6.75 for a four-group test (control, NAFLD, QHD treatment and GC treatment) based on expression levels of phosphorylated AMPK (fold-change = 2 between QHD treatment and NAFLD) in a similar study [14]. With an alpha = 0.05 and a power = 0.95, the projected sample size is 2 for each group. For an effect size (f = 1.75) that allows the same alpha and power in comparing the means of ~30% difference, the projected sample size is 3 for each group. We used relatively larger sample sizes (*N* = 6 ~ 10) in qRT-PCR and microbiome analysis, to achieve a similar statistical power for the comparison of means with small differences.

### Animals

The protocols for animal studies were reviewed and approved by the Animal Studies Ethics Committee of Shanghai University of Traditional Chinese Medicine and the Institutional Animal Care and Use Committee of University at Buffalo. For gene expression studies, four-week old male Sprague-Dawley rats or C57BL/6J mice, standard rodent chow (fat contributes 13.8% calories) and HFD (fat contributes 36.5% calories) were purchased from the Shanghai Laboratory Animal Center (Chinese Academy of Sciences, Shanghai, China). Animals were maintained at room temperature on a 12 h:12 h light-dark cycle in the animal center of the Shanghai University of Traditional Chinese Medicine. Rats and mice were randomized into four groups (*n* = 10 for each group). Animals in the: (i) control group were fed standard chow, (ii) NAFLD group were fed HFD, (iii) QHD group were fed HFD for four weeks followed by daily gavage of 0.1ml/kg QHD in addition to HFD for additional four weeks, and (iv) GC group were fed HFD for 4 weeks followed by daily gavage of a mixture of geniposide and chlorogenic acid (weight ratio=66.7:1) in addition to HFD for an additional four weeks. All rats were sacrificed for tissue collection at the end of the four-week therapy treatment. Preparation of QHD was done as previously described [14]. Briefly, Herba Artemisiae Capillaris, Rhizoma Polygoni Cuspidati and Rhizoma Curcumae Longae were extracted with ethanol. Herba Hyperici Japonici and Gardenia jasminoides ellis were extracted with water. Then the extracts were mixed and condensed to the density of 0.93g/ crude herb/ml. The dose for an average adult NAFLD patient is 56 g/day, which include Herba Artemisiae Capillaris 16g, Rhizoma Polygoni Cuspidati 12g, Herba Hyperici Japonici 8g, Rhizoma Curcumae Longae 12g and Gardenia jasminoides ellis 8g. No side effect was observed at this dose. Geniposide (purity > 98%) and chlorogenic acid (purity > 98%) were purchased from Shanghai Winherd medical technology Co., Ltd, Shanghai, China. Rats in the control and NAFLD groups were gavaged with water for four weeks. All rats in the study were fed ad lib and had unlimited access to water.

Because geniposide and chlorogenic acid appear to be the active ingredients of QHD, we focused on GC in the microbiome study. For the microbiome study, four-week old male Sprague-Dawley rats were purchased from the Harlan Laboratories (Indianapolis, IN) and were split into two groups: i) a control group, consisting of 10 rats fed HFD (TD.06414, Harlan Laboratories, Indianapolis, IN). For this group drinking water was supplemented with Dextran Sulphate Sodium (DSS, 1%) for 7 days, followed by a 10 day wash-out period, and then the process was repeated, and ii) a GC group, consisting of 10 rats fed HFD and DSS for 12 weeks. At the end of week 12, rats in the GC group were gavaged with GC while rats in the control group were gavaged with water. All rats were sacrificed for tissue collection after 13 weeks of GC therapy. Fat contributed 60% of calories in TD.06414 diet, compared to 18% in the standard rodent chow (2018s). DSS feeding causes increased gut permeability, which is a characteristic of NAFLD. With DSS feeding, it is more likely that the gut microbiome will have an impact on NAFLD livers through the portal circulation. We therefore chose this model of HFD plus DSS feeding for the microbiome study.

### Biochemical assays

Serum glucose, insulin, AST, ALT and GSH biochemical assays were done as described previously [57]. Triglyceride in rat liver and serum was determined using a colorimetric kit from Cayman Chemical Company (#10010303, Ann Arbor, MI) according to manufacturer's directions. Blood LPS was determined with the ToxinSensorTM Chromogenic Limulus Amebocyte Lysate (LAL) Endotoxin Assay Kit (GenScript, Piscataway, NJ, USA) according to manufacturer's directions.

### Histopathology

Liver and colon pieces of about 5mm in all dimensions were obtained from rats and fixed in 4% formaldehyde for 15 min. After fixation, specimens were sequentially equilibrated in 30% sucrose, 15% sucrose/50% Optimal Cutting Temperature medium (OCT, Sakura Finetek, Torrance, CA) and 100% OCT. Liver and colon pieces were subsequently frozen in OCT and 10 μm sections were cut with a cryostat. Sections were stained with hematoxylin/eosin (H&E) or oil red O and visualized by light microscopy.

### Microarray analysis

Total RNA was extracted from rat livers using RNeasy (Qiagen, Valencia, CA). RNA samples were subjected to transcriptome analysis using Affymetrix Rat Genome 230 2.0 Array. Microarray data is available at PubMed under accession number GSE87432. The MicroArray Suite 5 (MAS5) was used for data normalization in consideration that MAS5 makes use of the ‘Perfect Match – Mismatch’ signals and that expression values determined from MAS5 are not on logarithmic scale. Pathway analysis was performed with the Ingenuity Pathways Analysis (IPA; Ingenuity Systems, Inc., Redwood City, CA, www.ingenuity.com). Input lists included DEGs between experimental groups (False Discovery Rate < 0.05, *T* test).

### Quantitative real-time PCR (qRT-PCR)

Custom primers were designed using the NIH primer tool (http://www.ncbi.nlm.nih.gov/tools/primer-blast/), with a melting temperature of greater than 60°C, and separated by at least one intron of greater than 1000 nucleotides (Supplementary Table 7). Primers were characterized by melting curve analysis, agarose gel electrophoresis and DNA sequencing and synthesized at Eurofins MWG Operon (Huntsville, Alabama). Animal tissues were stored at −80 °C after soaking with RNAlater (Qiagen, Valencia, CA). Total RNA isolated using the RNeasy kit (Qiagen, Valencia, CA) was used to prepare cDNA with the i-Script cDNA synthesis kit (Bio-Rad Laboratories, Hercules, CA). Quantitative RT-PCR was performed on an iCycler iQ real-time detection system (Bio-Rad Laboratories) using SYBR Green (iQ SYBR Green Supermix, Bio-Rad Laboratories). Relative expression of each gene was calculated with GAPDH as the housekeeping gene as previously described [45].

### Microbiome analysis

Colon contents were collected and stored at −80°C before DNA isolation using the ZR Fecal DNA MicroPrep (Zymo Research, Irvine, CA). The targeted V3-V4 hypervariable region was amplified with primer pair (319F: 5′ACTCCTACGGGAGGCAGCAG 3′; 806R: 3′ACTCCTACGGGAGGCAGCAG 5′). Amplicons from all samples were multiplexed and paired-end sequenced on an Illumina Miseq at the Genomics Research Center of University of Rochester, following a published dual-indexing protocol [58].

Initial Illumina basecall raw data was processed into 2 × 300 FASTQ paired-end reads using Illumina's bcl2fastq (v1.8.4) without demultiplexing. The barcodes in each read of the paired sequencing reads were removed, concatenated together and stored in a separate file. Each pair of reads was then stitched together, with the combined barcodes attached. FASTQ format read files were then converted to FASTA and QUAL files and quality filtered using Quantitative Insights into Microbial Ecology (QIIME) software version 1.8.0 [59]. De novo OTU picking was performed with QIIME at a sequence similarity level of 97%, which approximates species-level phylotypes. Chimeric sequences were removed before taxonomy assignment using the RDP classifier, Pynast and a phylogenetic tree constructed with FastTree2. β-diversity between all tested samples was evaluated with UniFrac-based [60] principle coordinates analysis with a rarefaction depth of 39016 determined by the minimum OTU count for all samples.

### Additional statistical analysis

Student T tests or Mann-Whitney U tests with two-tailed distribution were performed to examine statistical differences between two experimental groups. One-way ANOVA (or Kruskal Walis test) was performed to compare the means of 3 or more groups, followed by post-hoc Tukey's test (or Dunn's multiple comparisons test) for pairwise comparisons. A *P*-value < 0.05 was considered to be significant.

## SUPPLEMENTARY MATERIALS FIGURES AND TABLES















## Abbreviations

DEG: differentially expressed gene
HFD: high-fat diet
LPS: Lipopolysaccharide
NAFLD: non-alcoholic fatty liver disease
NASH: non-alcoholic steatohepatitis
GC: geniposide and chlorogenic acid
OTU: operational taxonomic unit
QHD: Qushi Huayu Decoction
qRT-PCR: Quantitative Real-Time PCR
ROS: reactive oxygen species
