# Supplementary material for: Multi-targeting therapeutic mechanisms of the Chinese herbal medicine QHD in the treatment of non-alcoholic fatty liver disease
Source: Oncotarget. 2017 Feb 18;8(17):27820–38. doi: 10.18632/oncotarget.15482 (PMC5438611; doi:10.18632/oncotarget.15482)
Supplement: Supplementary file 1 [file oncotarget-08-27820-s001.pdf]

Multi-targeting therapeutic mechanisms of the Chinese herbal medicine QHD in the treatment of non-alcoholic fatty liver disease

Supplementary Materials

Supplementary Table 1: Pathways enriched with DEGs with elevated expression in NAFLD model compared to control (*P* value < 0.05). see Supplementary_Table_1

Supplementary Table 2: Pathways enriched with DEGs with decreased expression in NAFLD model compared to control (*P* value < 0.05)[a]

Ingenuity Canonical Pathways[b]	*P* value[c]	Molecules[d]
Adipogenesis pathway	0.0069	HDAC4,FZD3,SMO,FBXW7,RPS6KC1
Factors Promoting Cardiogenesis in Vertebrates	0.0110	FZD3,SMO,NPPA,TCF7L2
Oxidative Ethanol Degradation III	0.0120	ACSS2,ALDH3A1
Ethanol Degradation IV	0.0178	ACSS2,ALDH3A1
Tetrahydrobiopterin Biosynthesis I	0.0275	PTS
1D-myo-inositol Hexakisphosphate Biosynthesis V (from Ins(1,3,4)P3)	0.0275	ITPK1
Tetrahydrobiopterin Biosynthesis II	0.0275	PTS
Basal Cell Carcinoma Signaling	0.0302	FZD3,SMO,TCF7L2
Ovarian Cancer Signaling	0.0347	BRAF,FZD3,SMO,TCF7L2
Role of Wnt/GSK-3βSignaling in the Pathogenesis of Influenza	0.0355	FZD3,SMO,TCF7L2
Acetate Conversion to Acetyl-CoA	0.0372	ACSS2
Ethanol Degradation II	0.0427	ACSS2,ALDH3A1
HIPPO signaling	0.0468	TJP2,AJUBA,PARD3
Epithelial Adherens Junction Signaling	0.0490	MYH2,Actn3,PARD3,TCF7L2

[a]Pathway analysis was performed with Ingenuity Pathways Analysis (IPA; Ingenuity Systems, Inc., Redwood City, CA, www.ingenuity.com) tool. Canonical pathways with significant *p* values (*p* value < 0.05) are listed.
[b]Enriched canonical pathways associated with the input gene list.
[c]*P* values calculated by Fisher's exact test right-tailed for gene enrichment analysis, It ranges from 0 to 1.
Fisher's exact *P* Value = 0 represents perfect enrichment. P values smaller than 0.05 are considered strongly enriched in the canonical pathways.
[d]Molecules in the pathway overlapping with the input gene list.

Supplementary Table 3: Pathways enriched with DEGs with elevated expression in QHD compared to NAFLD model (*P* value < 0.05). see Supplementary_Table_3

Supplementary Table 4: Pathways enriched with DEGs with decreased expression in QHD compared to NAFLD model (*P* value < 0.05). see Supplementary_Table_4

Supplementary Table 5: Pathways enriched with DEGs with elevated expression in GC compared to NAFLD model (*P* value < 0.05). see Supplementary_Table_5

Supplementary Table 6: Pathways enriched with DEGs with decreased expression in GC compared to NAFLD model (*P* value < 0.05). see Supplementary_Table_6

Supplementary Table 7: Primers for qRT-PCR. see Supplementary_Table_7
