# Supplementary material for: Multi-targeting therapeutic mechanisms of the Chinese herbal medicine QHD in the treatment of non-alcoholic fatty liver disease
Source: Oncotarget. 2017 Feb 18;8(17):27820–38. doi: 10.18632/oncotarget.15482 (PMC5438611; doi:10.18632/oncotarget.15482)
Supplement: Supplementary file 2 [file oncotarget-08-27820-s002.docx]

Supplementary Table 1: Pathways enriched with DEGs with elevated expression in NAFLD model compared to control (P value < 0.05)^a^

| Ingenuity Canonical Pathways^b^ | P value^c^ | Molecules^d^ |
| --- | --- | --- |
| Molecular Mechanisms of Cancer | <0.0001 | CDK4,NFKB1,XIAP,SYNGAP1,PAK1,Pak2,APH1A,PRKCD,PAK2,ADCY1,BID,CFLAR,BRCA1,CHEK2,BCL2L11,CDK2,PRKCB |
| Natural Killer Cell Signaling | <0.0001 | PTPN6,PAK1,TYROBP,PRKCD,Pak2,PAK2,FCGR3A/FCGR3B,INPP5D,PRKCB |
| Pyridoxal 5'-phosphate Salvage Pathway | <0.0001 | PRKX,PAK1,PRKCD,Pak2,PAK2,CDK4,CDK2 |
| TNFR1 Signaling | <0.0001 | PAK1,Pak2,PAK2,BID,NFKB1,XIAP |
| Renin-Angiotensin Signaling | <0.0001 | PTPN6,PAK1,PRKCD,Pak2,ADCY1,PAK2,NFKB1,PRKCB |
| Salvage Pathways of Pyrimidine Ribonucleotides | 0.0001 | PRKX,PAK1,PRKCD,Pak2,PAK2,CDK4,CDK2 |
| TREM1 Signaling | 0.0002 | SIGIRR,IL18,TYROBP,TLR1,CD86,NFKB1 |
| CD28 Signaling in T Helper Cells | 0.0004 | PTPN6,PAK1,CD3E,BCL10,HLA-DRA,CD86,NFKB1 |
| Fcγ Receptor-mediated Phagocytosis in Macrophages and Monocytes | 0.0007 | PLD4,PAK1,PRKCD,FCGR3A/FCGR3B,INPP5D,PRKCB |
| GNRH Signaling | 0.0007 | PAK1,PRKCD,Pak2,ADCY1,PAK2,NFKB1,PRKCB |
| Hereditary Breast Cancer Signaling | 0.0007 | UBD,HDAC11,MSH6,CDK4,BRCA1,CHEK2,MLH1 |
| Mechanisms of Viral Exit from Host Cells | 0.0012 | PRKCD,VPS36,LMNB1,PRKCB |
| GADD45 Signaling | 0.0012 | CDK4,BRCA1,CDK2 |
| IL-3 Signaling | 0.0013 | PTPN6,PAK1,PRKCD,INPP5D,PRKCB |
| Toll-like Receptor Signaling | 0.0015 | SIGIRR,UBD,IL18,TLR1,NFKB1 |
| ErbB Signaling | 0.0030 | PAK1,PRKCD,Pak2,PAK2,PRKCB |
| Altered T Cell and B Cell Signaling in Rheumatoid Arthritis | 0.0033 | IL18,HLA-DRA,TLR1,CD86,NFKB1 |
| Cell Cycle Control of Chromosomal Replication | 0.0035 | CDK4,CHEK2,CDK2 |
| Crosstalk between Dendritic Cells and Natural Killer Cells | 0.0035 | IL18,TYROBP,HLA-DRA,CD86,NFKB1 |
| Cardiacβ-adrenergic Signaling | 0.0042 | PPP1R14C,ADRBK2,PKIB,ADCY1,PDE4B,MPPE1 |
| Prostanoid Biosynthesis | 0.0044 | PTGS1,HPGDS |
| ATM Signaling | 0.0046 | BID,BRCA1,CHEK2,CDK2 |
| Protein Kinase A Signaling | 0.0048 | PPP1R14C,PTPN6,DUSP5,PRKCD,ADCY1,PTPN1,PTPN18,PDE4B,NFKB1,MPPE1,PRKCB |
| Calcium-induced T Lymphocyte Apoptosis | 0.0060 | CD3E,PRKCD,HLA-DRA,PRKCB |
| B Cell Development | 0.0062 | IL7R,HLA-DRA,CD86 |
| TWEAK Signaling | 0.0068 | BID,NFKB1,XIAP |
| Angiopoietin Signaling | 0.0068 | PAK1,Pak2,PAK2,NFKB1 |
| Erythropoietin Signaling | 0.0071 | PTPN6,PRKCD,NFKB1,PRKCB |
| iCOS-iCOSL Signaling in T Helper Cells | 0.0078 | CD3E,HLA-DRA,ICOS,NFKB1,INPP5D |
| CXCR4 Signaling | 0.0079 | PAK1,PRKCD,Pak2,ADCY1,PAK2,PRKCB |
| phagosome formation | 0.0081 | PRKCD,TLR1,FCGR3A/FCGR3B,INPP5D,PRKCB |
| Type I Diabetes Mellitus Signaling | 0.0085 | CD3E,HLA-DRA,CD86,BID,NFKB1 |
| T Helper Cell Differentiation | 0.0087 | IL18,HLA-DRA,ICOS,CD86 |
| Renal Cell Carcinoma Signaling | 0.0087 | UBD,PAK1,Pak2,PAK2 |
| Small Cell Lung Cancer Signaling | 0.0087 | CDK4,BID,NFKB1,CDK2 |
| Tec Kinase Signaling | 0.0091 | PAK1,PRKCD,Pak2,PAK2,NFKB1,PRKCB |
| Guanosine Nucleotides Degradation III | 0.0093 | PNP,ACPP |
| LPS-stimulated MAPK Signaling | 0.0095 | PAK1,PRKCD,NFKB1,PRKCB |
| Urate Biosynthesis/Inosine 5'-phosphate Degradation | 0.0107 | PNP,ACPP |
| CCR3 Signaling in Eosinophils | 0.0110 | PAK1,PRKCD,Pak2,PAK2,PRKCB |
| PKCθ Signaling in T Lymphocytes | 0.0112 | CD3E,BCL10,HLA-DRA,CD86,NFKB1 |
| Xanthine and Xanthosine Salvage | 0.0115 | PNP |
| Systemic Lupus Erythematosus Signaling | 0.0117 | IL18,PTPN6,CD3E,PRPF8,CD86,FCGR3A/FCGR3B,INPP5D |
| Role of BRCA1 in DNA Damage Response | 0.0120 | MSH6,BRCA1,CHEK2,MLH1 |
| Granzyme B Signaling | 0.0138 | BID,LMNB1 |
| Mismatch Repair in Eukaryotes | 0.0138 | MSH6,MLH1 |
| NF-κB Signaling | 0.0145 | SIGIRR,IL18,BCL10,TLR1,NFKB1,PRKCB |
| Role of Pattern Recognition Receptors in Recognition of Bacteria and Viruses | 0.0145 | IL18,PRKCD,TLR1,NFKB1,PRKCB |
| B Cell Receptor Signaling | 0.0148 | PTPN6,DAPP1,BCL10,NFKB1,INPP5D,PRKCB |
| PI3K Signaling in B Lymphocytes | 0.0155 | DAPP1,BCL10,NFKB1,INPP5D,PRKCB |
| Adenosine Nucleotides Degradation II | 0.0155 | PNP,ACPP |
| Dendritic Cell Maturation | 0.0158 | IL18,TYROBP,HLA-DRA,CD86,NFKB1,FCGR3A/FCGR3B |
| Ovarian Cancer Signaling | 0.0170 | PTGS1,MSH6,CDK4,BRCA1,MLH1 |
| Primary Immunodeficiency Signaling | 0.0174 | IL7R,CD3E,ICOS |
| Graft-versus-Host Disease Signaling | 0.0174 | IL18,HLA-DRA,CD86 |
| CTLA4 Signaling in Cytotoxic T Lymphocytes | 0.0182 | PTPN6,CD3E,CD86,AP1G1 |
| G Beta Gamma Signaling | 0.0182 | PAK1,PRKCD,ADCY1,PRKCB |
| Communication between Innate and Adaptive Immune Cells | 0.0186 | IL18,HLA-DRA,TLR1,CD86 |
| Apoptosis Signaling | 0.0186 | BID,NFKB1,BCL2L11,XIAP |
| Phospholipase C Signaling | 0.0191 | PLD4,CD3E,HDAC11,PRKCD,ADCY1,NFKB1,PRKCB |
| DNA damage-induced 14-3-3_ Signaling | 0.0195 | BRCA1,CDK2 |
| ERK/MAPK Signaling | 0.0204 | PPP1R14C,PAK1,PRKCD,Pak2,PAK2,PRKCB |
| Death Receptor Signaling | 0.0209 | BID,CFLAR,NFKB1,XIAP |
| Purine Nucleotides Degradation II (Aerobic) | 0.0214 | PNP,ACPP |
| RAR Activation | 0.0219 | PRKCD,ADCY1,ERCC2,NFKB1,PPARGC1A,PRKCB |
| Breast Cancer Regulation by Stathmin1 | 0.0224 | PPP1R14C,PAK1,PRKCD,ADCY1,CDK2,PRKCB |
| Semaphorin Signaling in Neurons | 0.0224 | PAK1,Pak2,PAK2 |
| Guanine and Guanosine Salvage I | 0.0224 | PNP |
| Adenine and Adenosine Salvage I | 0.0224 | PNP |
| Role of CHK Proteins in Cell Cycle Checkpoint Control | 0.0245 | BRCA1,CHEK2,CDK2 |
| p53 Signaling | 0.0257 | CDK4,BRCA1,CHEK2,CDK2 |
| 3-phosphoinositide Degradation | 0.0263 | PTPN6,PPM1H,MTMR14,PTPN1,INPP5D |
| Nur77 Signaling in T Lymphocytes | 0.0269 | CD3E,HLA-DRA,CD86 |
| G-Protein Coupled Receptor Signaling | 0.0275 | SYNGAP1,PTGDR,ADCY1,PDE4B,NFKB1,MPPE1,PRKCB |
| Role of JAK1, JAK2 and TYK2 in Interferon Signaling | 0.0302 | PTPN6,NFKB1 |
| Estrogen-mediated S-phase Entry | 0.0302 | CDK4,CDK2 |
| Induction of Apoptosis by HIV1 | 0.0309 | BID,NFKB1,XIAP |
| ErbB4 Signaling | 0.0309 | APH1A,PRKCD,PRKCB |
| Rac Signaling | 0.0309 | PAK1,Pak2,PAK2,NFKB1 |
| HGF Signaling | 0.0316 | PAK1,PRKCD,CDK2,PRKCB |
| Pancreatic Adenocarcinoma Signaling | 0.0331 | PLD4,CDK4,NFKB1,CDK2 |
| 5-aminoimidazole Ribonucleotide Biosynthesis I | 0.0339 | PFAS |
| Cell Cycle: G1/S Checkpoint Regulation | 0.0363 | HDAC11,CDK4,CDK2 |
| Eicosanoid Signaling | 0.0363 | PTGDR,PTGS1,HPGDS |
| Hepatic Cholestasis | 0.0380 | IL18,PRKCD,ADCY1,NFKB1,PRKCB |
| Androgen Signaling | 0.0380 | PRKCD,ERCC2,NFKB1,PRKCB |
| LPS/IL-1 Mediated Inhibition of RXR Function | 0.0398 | IL18,MGMT,PPARGC1B,FMO4,ABCA1,PPARGC1A |
| GABA Receptor Signaling | 0.0407 | UBD,KCNN4,ADCY1 |
| Cdc42 Signaling | 0.0427 | PAK1,CD3E,HLA-DRA,PAK2,EXOC6 |
| Macropinocytosis Signaling | 0.0427 | PAK1,PRKCD,PRKCB |
| TNFR2 Signaling | 0.0427 | NFKB1,XIAP |
| CCR5 Signaling in Macrophages | 0.0437 | CD3E,PRKCD,PRKCB |
| Agrin Interactions at Neuromuscular Junction | 0.0437 | PAK1,Pak2,PAK2 |
| Growth Hormone Signaling | 0.0437 | PTPN6,PRKCD,PRKCB |
| Arsenate Detoxification I (Glutaredoxin) | 0.0447 | PNP |
| Spermine and Spermidine Degradation I | 0.0447 | SAT1 |
| N-acetylglucosamine Degradation II | 0.0447 | NAGK |
| Role of NFAT in Regulation of the Immune Response | 0.0457 | CD3E,HLA-DRA,CD86,NFKB1,FCGR3A/FCGR3B |
| Endothelin-1 Signaling | 0.0468 | PLD4,PRKCD,PTGS1,ADCY1,PRKCB |
| Synaptic Long Term Potentiation | 0.0468 | PPP1R14C,PRKCD,ADCY1,PRKCB |
| P2Y Purigenic Receptor Signaling Pathway | 0.0468 | PRKCD,ADCY1,NFKB1,PRKCB |
| phagosome maturation | 0.0479 | HLA-DRA,Atp6ap1l,LAMP1,ATP6V0E1 |
| JAK/Stat Signaling | 0.0490 | PTPN6,PTPN1,NFKB1 |
| Gustation Pathway | 0.0490 | ADCY1,PDE4B,PANX1,MPPE1 |

^a^Pathway analysis was performed with Ingenuity Pathways Analysis ( IPA; Ingenuity Systems, Inc., Redwood City, CA, www.ingenuity.com) tool. Canonical pathways with significant p values (p value < 0.05) are listed.

^b^Enriched canonical pathways associated with the input gene list.

^c^P values calculated by Fisher's exact test right-tailed for gene enrichment analysis, It ranges from 0 to 1. Fisher's exact P Value = 0 represents perfect enrichment. P values smaller than 0.05 are considered strongly enriched in the canonical pathways.

^d^Molecules in the pathway overlapping with the input gene list.
