# Supplementary material for: Multi-targeting therapeutic mechanisms of the Chinese herbal medicine QHD in the treatment of non-alcoholic fatty liver disease
Source: Oncotarget. 2017 Feb 18;8(17):27820–38. doi: 10.18632/oncotarget.15482 (PMC5438611; doi:10.18632/oncotarget.15482)
Supplement: Supplementary file 3 [file oncotarget-08-27820-s003.docx]

Supplementary Table 3: Pathways enriched with DEGs with elevated expression in QHD compared to NAFLD model (P value < 0.05)^a^

| Ingenuity Canonical Pathways^b^ | P value^c^ | Molecules^d^ |
| --- | --- | --- |
| Mitochondrial Dysfunction | 0.0007 | SDHB,NDUFA9,PRDX5,ACO2,UQCR11,NDUFB8,GSR,ATP5C1,NDUFS1,PARK7,UQCRFS1,SDHD,HTRA2,AIFM1 |
| Methylglyoxal Degradation III | 0.0011 | AKR7A2,AKR7A3,AKR1A1,ADH4 |
| Glutathione-mediated Detoxification | 0.0015 | NAT8,MGST2,GSTM5,Gstt1,GSTO1 |
| Protein Ubiquitination Pathway | 0.0019 | PSMB3,PSMB4,USP24,PSMA6,PSMB5,USP15,HLA-A,PSMD13,HSPA1A/HSPA1B,USP9X,PSMC4,USP39,PSMD8,PSMB2,PSMD2,PSMA4,PSMD4 |
| Heme Biosynthesis II | 0.0019 | UROD,FECH,ALAS1 |
| Glutathione Redox Reactions I | 0.0022 | GSR,MGST2,Gstt1,PRDX6 |
| Glutathione Biosynthesis | 0.0026 | GCLC,GCLM |
| D-glucuronate Degradation I | 0.0026 | AKR1A1,CRYL1 |
| Aryl Hydrocarbon Receptor Signaling | 0.0033 | MYC,ALDH1L1,CYP1A1,ALDH1A1,CYP1A2,GSTM5,MGST2,CDK6,ATR,DHFR,GSTO1 |
| Ethanol Degradation II | 0.0037 | AKR1A1,ADH7,ALDH1A1,ADH1C,ADH4 |
| TCA Cycle II (Eukaryotic) | 0.0045 | SDHB,ACO2,SDHD,IDH3B |
| Heme Biosynthesis from Uroporphyrinogen-III I | 0.0051 | UROD,FECH |
| Proline Biosynthesis I | 0.0051 | PYCR2,PYCR1 |
| Noradrenaline and Adrenaline Degradation | 0.0052 | AKR1A1,ADH7,ALDH1A1,ADH1C,ADH4 |
| Hereditary Breast Cancer Signaling | 0.0055 | NPM1,FANCB,POLR2F,RRAS,POLR2E,H2AFX,WEE1,CDK6,ATR,DDB2 |
| tRNA Charging | 0.0059 | TARS2,YARS2,FARS2,DARS,SARS |
| Xenobiotic Metabolism Signaling | 0.0079 | ABCB1,ALDH1L1,CYP1A1,CYP1A2,ALDH1A1,MGST2,UGT2B17,GSTM5,RRAS,CES5A,GCLC,UGT2B11,ABCC3,FMO4,GSTO1,ESD |
| NRF2-mediated Oxidative Stress Response | 0.0083 | AKR7A2,GSR,AKR7A3,AKR1A1,MGST2,GSTM5,RRAS,GCLC,AOX1,GCLM,GSTO1,EPHX1 |
| dTMP De Novo Biosynthesis | 0.0085 | DHFR,SHMT2 |
| Folate Polyglutamylation | 0.0085 | MTHFD1,SHMT2 |
| γ-glutamyl Cycle | 0.0093 | GGCT,GCLC,GCLM |
| Nicotine Degradation II | 0.0115 | ADH7,CYP1A1,CYP1A2,UGT2B17,AOX1,FMO4 |
| Proline Biosynthesis II (from Arginine) | 0.0123 | PYCR2,PYCR1 |
| Arginine Degradation VI (Arginase 2 Pathway) | 0.0123 | PYCR2,PYCR1 |
| PXR/RXR Activation | 0.0151 | ABCB1,ALDH1A1,CYP1A2,PRKAR2A,ALAS1,ABCC3 |
| Serotonin Degradation | 0.0151 | AKR1A1,ADH7,ALDH1A1,UGT2B17,ADH1C,ADH4 |
| Retinoate Biosynthesis I | 0.0166 | ADH7,ALDH1A1,ADH1C,ADH4 |
| Oxidative Phosphorylation | 0.0170 | ATP5C1,SDHB,NDUFS1,NDUFA9,UQCRFS1,SDHD,NDUFB8,UQCR11 |
| STAT3 Pathway | 0.0224 | MYC,PTPN2,RRAS,FGFR2,IGF2R,SOCS5 |
| Nicotine Degradation III | 0.0224 | ADH7,CYP1A1,CYP1A2,UGT2B17,AOX1 |
| Folate Transformations I | 0.0282 | MTHFD1,SHMT2 |
| Netrin Signaling | 0.0288 | UNC5B,PRKAR2A,ABLIM1,NTN1 |
| Phospholipases | 0.0295 | PNPLA8,PLD2,PLB1,PLA1A,PRDX6 |
| LPS/IL-1 Mediated Inhibition of RXR Function | 0.0331 | IL1R2,ABCB1,ALDH1L1,ALDH1A1,MGST2,GSTM5,ALAS1,ABCC3,FMO4,IL1RAP,GSTO1,IL36B |
| 3-phosphoinositide Degradation | 0.0331 | MTMR6,PTPN2,PTPN13,PDXP,MTMR14,MTMR2,NUDT2,PPP1CA,NUDT1 |
| Purine Nucleotides De Novo Biosynthesis II | 0.0417 | ADSL,GMPS |
| D-myo-inositol (1,4,5,6)-Tetrakisphosphate Biosynthesis | 0.0427 | MTMR6,PTPN2,PTPN13,PDXP,MTMR2,NUDT2,PPP1CA,NUDT1 |
| D-myo-inositol (3,4,5,6)-tetrakisphosphate Biosynthesis | 0.0427 | MTMR6,PTPN2,PTPN13,PDXP,MTMR2,NUDT2,PPP1CA,NUDT1 |
| Role of Tissue Factor in Cancer | 0.0479 | MTOR,P4HB,RRAS,PDXP,ITGAV,HBEGF,LIMK2 |
| 3-phosphoinositide Biosynthesis | 0.0490 | MTMR6,PTPN2,PTPN13,PDXP,MTMR2,NUDT2,PPP1CA,PI4KA,NUDT1 |

^a^Pathway analysis was performed with Ingenuity Pathways Analysis ( IPA; Ingenuity Systems, Inc., Redwood City, CA, www.ingenuity.com) tool. Canonical pathways with significant p values (p value < 0.05) are listed.

^b^Enriched canonical pathways associated with the input gene list.

^c^P values calculated by Fisher's exact test right-tailed for gene enrichment analysis, It ranges from 0 to 1. Fisher's exact P Value = 0 represents perfect enrichment. P values smaller than 0.05 are considered strongly enriched in the canonical pathways.

^d^Molecules in the pathway overlapping with the input gene list.
