# Supplementary material for: Multi-targeting therapeutic mechanisms of the Chinese herbal medicine QHD in the treatment of non-alcoholic fatty liver disease
Source: Oncotarget. 2017 Feb 18;8(17):27820–38. doi: 10.18632/oncotarget.15482 (PMC5438611; doi:10.18632/oncotarget.15482)
Supplement: Supplementary file 4 [file oncotarget-08-27820-s004.docx]

Supplementary Table 4: Pathways enriched with DEGs with decreased expression in QHD compared to NAFLD model (P value < 0.05)^a^

| Ingenuity Canonical Pathways^b^ | P value^c^ | Molecules^d^ |
| --- | --- | --- |
| Ephrin A Signaling | 0.0001 | EPHA8,EFNA5,PIK3C2G,EPHA5,EPHA2,LIMK1,EFNA1 |
| Cysteine Biosynthesis/Homocysteine Degradation | 0.0005 | CBS/CBSL,CTH |
| L-cysteine Degradation I | 0.0032 | CDO1,GOT2 |
| G Beta Gamma Signaling | 0.0046 | CAV2,ADCY1,GNAZ,PRKD3,DNM2,EGFR,PRKAR1A |
| Superpathway of Methionine Degradation | 0.0063 | CBS/CBSL,CTH,CDO1,GOT2 |
| p70S6K Signaling | 0.0069 | F2RL3,PPP2R4,IRS1,PPP2R2B,PIK3C2G,PRKD3,AGTR1,EGFR |
| Glioma Signaling | 0.0069 | CAMK2D,IGF1,PDGFRA,PIK3C2G,PRKD3,PDGFRB,EGFR |
| Ephrin Receptor Signaling | 0.0081 | EPHA8,ANGPT1,EFNA5,CXCL12,PIK3C2G,EPHA5,GNAZ,EPHA2,LIMK1,EFNA1 |
| Cell Cycle Regulation by BTG Family Proteins | 0.0087 | PPP2R4,PPP2R2B,BTG1,CDK2 |
| RAR Activation | 0.0145 | RXRG,PNRC1,RARA,RARB,ADCY1,IGFBP3,TGFB3,PRKD3,CRABP1,PRKAR1A |
| Retinoic acid Mediated Apoptosis Signaling | 0.0148 | RXRG,RARA,RARB,TNKS2,CRABP1 |
| Tight Junction Signaling | 0.0170 | CLDN1,PPP2R4,HSF1,PVRL3,VAPA,PPP2R2B,TGFB3,PVRL2,PRKAR1A |
| Wnt/β-catenin Signaling | 0.0182 | AXIN2,LRP5,SOX6,PPP2R4,RARA,PPP2R2B,RARB,TGFB3,WNT4 |
| eNOS Signaling | 0.0186 | GUCY1A3,ADCY1,PIK3C2G,CHRNE,PRKD3,DNM2,PRKAR1A,Gucy1b2 |
| Ketogenesis | 0.0219 | BDH1,HMGCS1 |
| Glycine Betaine Degradation | 0.0219 | SRR,DMGDH |
| Growth Hormone Signaling | 0.0229 | IGF1,IRS1,IGFBP3,PIK3C2G,PRKD3 |
| L-cysteine Degradation II | 0.0234 | CTH |
| Citrulline Degradation | 0.0234 | OTC |
| Small Cell Lung Cancer Signaling | 0.0251 | PIAS3,RXRG,RARB,PIK3C2G,CDK2 |
| Molecular Mechanisms of Cancer | 0.0257 | LRP5,TAB2,PIK3C2G,GNAZ,BMP5,XIAP,CTNNA2,CAMK2D,IRS1,ADCY1,TGFB3,WNT4,PRKD3,CDK2,PRKAR1A |
| Axonal Guidance Signaling | 0.0275 | NFATC3,PIK3C2G,CXCL12,GNAZ,BMP5,EFNA1,LIMK1,EPHA8,IGF1,EFNA5,RTN4,WNT4,EPHA5,SEMA4G,PRKD3,EPHA2,PRKAR1A |
| STAT3 Pathway | 0.0282 | PIAS3,FGFR4,PDGFRA,PDGFRB,EGFR |
| Hepatic Fibrosis / Hepatic Stellate Cell Activation | 0.0288 | COL5A1,FN1,IGF1,PDGFRA,IGFBP3,TGFB3,AGTR1,PDGFRB,EGFR |
| cAMP-mediated signaling | 0.0339 | CAMK2D,VIPR1,CREM,PTH1R,ADCY1,HRH3,AGTR1,MPPE1,PRKAR1A,OPRL1 |
| Dopamine-DARPP32 Feedback in cAMP Signaling | 0.0363 | GUCY1A3,PPP2R4,CREM,PPP2R2B,ADCY1,PRKD3,PRKAR1A,Gucy1b2 |
| VDR/RXR Activation | 0.0363 | RXRG,LRP5,IGFBP3,HES1,PRKD3 |
| Dopamine Receptor Signaling | 0.0363 | PPP2R4,PPP2R2B,ADCY1,SLC18A2,PRKAR1A |
| Breast Cancer Regulation by Stathmin1 | 0.0363 | CAMK2D,PPP2R4,PPP2R2B,ADCY1,PIK3C2G,PRKD3,CDK2,LIMK1,PRKAR1A |
| Human Embryonic Stem Cell Pluripotency | 0.0380 | FGFR4,PDGFRA,PIK3C2G,TGFB3,WNT4,BMP5,PDGFRB |
| Role of CHK Proteins in Cell Cycle Checkpoint Control | 0.0398 | PPP2R4,PPP2R2B,TLK2,CDK2 |
| Relaxin Signaling | 0.0398 | GUCY1A3,ADCY1,PIK3C2G,GNAZ,MPPE1,PRKAR1A,Gucy1b2 |
| Phenylalanine Degradation IV (Mammalian, via Side Chain) | 0.0417 | ALDH2,GOT2 |
| Leukocyte Extravasation Signaling | 0.0437 | CTNNA2,TXK,CLDN1,MMP14,CXCL12,PIK3C2G,ARHGAP35,PRKD3,DLC1 |
| Taurine Biosynthesis | 0.0468 | CDO1 |

^a^Pathway analysis was performed with Ingenuity Pathways Analysis ( IPA; Ingenuity Systems, Inc., Redwood City, CA, www.ingenuity.com) tool. Canonical pathways with significant p values (p value < 0.05) are listed.

^b^Enriched canonical pathways associated with the input gene list.

^c^P values calculated by Fisher's exact test right-tailed for gene enrichment analysis, It ranges from 0 to 1. Fisher's exact P Value = 0 represents perfect enrichment. P values smaller than 0.05 are considered strongly enriched in the canonical pathways.

^d^Molecules in the pathway overlapping with the input gene list.
