# Supplementary material for: Multi-targeting therapeutic mechanisms of the Chinese herbal medicine QHD in the treatment of non-alcoholic fatty liver disease
Source: Oncotarget. 2017 Feb 18;8(17):27820–38. doi: 10.18632/oncotarget.15482 (PMC5438611; doi:10.18632/oncotarget.15482)
Supplement: Supplementary file 5 [file oncotarget-08-27820-s005.docx]

Supplementary Table 5, Pathways enriched with DEGs with elevated expression in GC compared to NAFLD model (P value < 0.05)^a^

| Ingenuity Canonical Pathways^b^ | P value^c^ | Molecules^d^ |
| --- | --- | --- |
| CXCR4 Signaling | 0.0015 | GNAI2,GNB3,RRAS,CD4,CXCL12,RHOJ,ARHGEF11,GNAZ,ELK1,ELMO1,PRKCB |
| D-glucuronate Degradation I | 0.0018 | AKR1A1,CRYL1 |
| Axonal Guidance Signaling | 0.0032 | FES,RRAS,COPS5,CXCL12,LIMK2,GNAZ,NTN1,GNAI2,KIF7,GNB3,NTRK3,ADAM10,PRKAG2,EPHB3,SRGAP2,ARHGEF11,GLI1,WNT5B,WNT11,WNT5A,PRKCB |
| Chemokine Signaling | 0.0085 | GNAI2,RRAS,CXCL12,LIMK2,CCL5,PRKCB |
| Basal Cell Carcinoma Signaling | 0.0091 | KIF7,GLI1,WNT5B,WNT11,TCF7L2,WNT5A |
| Ephrin B Signaling | 0.0098 | GNAI2,GNB3,CBL,CXCL12,EPHB3,GNAZ |
| Molecular Mechanisms of Cancer | 0.0107 | RRAS,SMAD7,CDK6,RHOJ,MDM2,GNAZ,XIAP,GNAI2,CBL,PRKAG2,ARHGEF11,ELK1,GLI1,WNT11,WNT5B,PRKCB,WNT5A |
| Glutathione Redox Reactions I | 0.0112 | GSR,Gstt1,PRDX6 |
| Protein Ubiquitination Pathway | 0.0120 | ANAPC2,PSMA6,PSMD13,PSMC4,MDM2,USP39,ANAPC1,XIAP,UCHL1,CBL,PSMA4,USP46,AMFR |
| Protein Kinase A Signaling | 0.0178 | ANAPC2,YWHAB,ANAPC7,PDE4B,ANAPC1,NTN1,GNAI2,PTPRH,AKAP14,GNB3,RHO,PRKAG2,PGP,DUSP18,ELK1,TCF7L2,PRKCB |
| GM-CSF Signaling | 0.0191 | CSF2RB,RRAS,CISH,ELK1,PRKCB |
| G Beta Gamma Signaling | 0.0229 | GNAI2,GNB3,RRAS,PRKAG2,GNAZ,PRKCB |
| Chondroitin Sulfate Biosynthesis (Late Stages) | 0.0275 | SULT4A1,CHPF,HS3ST2,HS3ST6 |
| CCR5 Signaling in Macrophages | 0.0288 | GNAI2,GNB3,CD4,CCL5,PRKCB |
| Cell Cycle Control of Chromosomal Replication | 0.0288 | ORC3,CDK6,ORC4 |
| Gαi Signaling | 0.0309 | GNAI2,GABBR2,LTB4R,GNB3,RRAS,PRKAG2,HRH3 |
| Ephrin Receptor Signaling | 0.0309 | GNAI2,GNB3,RRAS,GRIN2D,ADAM10,CXCL12,EPHB3,LIMK2,GNAZ |
| Granulocyte Adhesion and Diapedesis | 0.0339 | GNAI2,CLDN11,SELP,JAM3,CXCL12,SDC3,CCL5,HRH3,GLG1 |
| Cleavage and Polyadenylation of Pre-mRNA | 0.0347 | CSTF2,WDR33 |
| BER pathway | 0.0347 | PNKP,FEN1 |
| STAT3 Pathway | 0.0355 | PIAS3,RRAS,NTRK3,CISH,FGFR2 |
| Heparan Sulfate Biosynthesis (Late Stages) | 0.0355 | SULT4A1,HS3ST2,HS3ST6,PRDX6 |
| HER-2 Signaling in Breast Cancer | 0.0417 | RRAS,CDK6,MDM2,PARD3,PRKCB |
| Semaphorin Signaling in Neurons | 0.0427 | FES,DPYSL4,RHOJ,LIMK2 |
| Phototransduction Pathway | 0.0427 | GNB3,RHO,PRKAG2,OPN3 |
| Role of Wnt/GSK-3β Signaling in the Pathogenesis of Influenza | 0.0437 | CSNK1E,WNT5B,WNT11,TCF7L2,WNT5A |
| Chondroitin Sulfate Biosynthesis | 0.0457 | SULT4A1,CHPF,HS3ST2,HS3ST6 |
| Retinoate Biosynthesis I | 0.0490 | DHRS3,DHRS7C,RDH12 |
| Choline Degradation I | 0.0490 | CHDH |
| Sulfate Activation for Sulfonation | 0.0490 | PAPSS1 |

^a^Pathway analysis was performed with Ingenuity Pathways Analysis ( IPA; Ingenuity Systems, Inc., Redwood City, CA, www.ingenuity.com) tool. Canonical pathways with significant p values (p value < 0.05) are listed.

^b^Enriched canonical pathways associated with the input gene list.

^c^P values calculated by Fisher's exact test right-tailed for gene enrichment analysis, It ranges from 0 to 1. Fisher's exact P Value = 0 represents perfect enrichment. P values smaller than 0.05 are considered strongly enriched in the canonical pathways.

^d^Molecules in the pathway overlapping with the input gene list.
