# Supplementary material for: Multi-targeting therapeutic mechanisms of the Chinese herbal medicine QHD in the treatment of non-alcoholic fatty liver disease
Source: Oncotarget. 2017 Feb 18;8(17):27820–38. doi: 10.18632/oncotarget.15482 (PMC5438611; doi:10.18632/oncotarget.15482)
Supplement: Supplementary file 6 [file oncotarget-08-27820-s006.docx]

Supplementary Table 6, Pathways enriched with DEGs with decreased expression in GC compared to NAFLD model (P value < 0.05)^a^

| Ingenuity Canonical Pathways^b^ | P value^c^ | Molecules^d^ |
| --- | --- | --- |
| Cholesterol Biosynthesis I | 0.0009 | EBP,NSDHL,SC5D |
| Cholesterol Biosynthesis II (via 24,25-dihydrolanosterol) | 0.0009 | EBP,NSDHL,SC5D |
| Cholesterol Biosynthesis III (via Desmosterol) | 0.0009 | EBP,NSDHL,SC5D |
| Lysine Degradation II | 0.0021 | AASS,ALDH7A1 |
| IGF-1 Signaling | 0.0033 | IGF1,IGFBP3,PIK3C2G,IRS2,IGFBP1,PRKAR1A |
| Citrulline Biosynthesis | 0.0059 | LOC102724788/PRODH,OTC |
| Superpathway of Cholesterol Biosynthesis | 0.0081 | EBP,NSDHL,SC5D |
| p70S6K Signaling | 0.0089 | PLCE1,F2RL3,PIK3C2G,BCAP31,AGTR1,EGFR |
| Histidine Degradation VI | 0.0093 | UROC1,MICAL2 |
| Fatty Acid β-oxidation I | 0.0117 | SLC27A5,ACSL5,HSD17B8 |
| Ovarian Cancer Signaling | 0.0138 | VEGFA,BRAF,DVL1,PIK3C2G,EGFR,PRKAR1A |
| Estrogen-Dependent Breast Cancer Signaling | 0.0145 | IGF1,PIK3C2G,HSD17B8,EGFR |
| Citrulline Degradation | 0.0148 | OTC |
| Fatty Acid Activation | 0.0155 | SLC27A5,ACSL5 |
| Amyotrophic Lateral Sclerosis Signaling | 0.0158 | VEGFA,CAPN8,IGF1,PIK3C2G,GRIK1 |
| Non-Small Cell Lung Cancer Signaling | 0.0162 | FHIT,RARB,PIK3C2G,EGFR |
| Nitric Oxide Signaling in the Cardiovascular System | 0.0170 | VEGFA,GUCY1A3,PIK3C2G,ATP2A2,PRKAR1A |
| Sertoli Cell-Sertoli Cell Junction Signaling | 0.0178 | CTNNA2,GUCY1A3,PVRL3,SPTB,VCL,CLDN7,PRKAR1A |
| PXR/RXR Activation | 0.0178 | G6PC,IGFBP1,PAPSS2,PRKAR1A |
| Superpathway of Citrulline Metabolism | 0.0178 | LOC102724788/PRODH,OTC |
| Hepatic Fibrosis / Hepatic Stellate Cell Activation | 0.0204 | VEGFA,IGF1,LEPR,IGFBP3,AGTR1,COL3A1,EGFR |
| Melatonin Signaling | 0.0204 | BRAF,PLCE1,RORA,PRKAR1A |
| Histamine Degradation | 0.0234 | ALDH2,ALDH7A1 |
| Leptin Signaling in Obesity | 0.0245 | PLCE1,LEPR,PIK3C2G,PRKAR1A |
| γ-linolenate Biosynthesis II (Animals) | 0.0263 | SLC27A5,ACSL5 |
| Mitochondrial L-carnitine Shuttle Pathway | 0.0263 | SLC27A5,ACSL5 |
| D-myo-inositol (1,4,5)-trisphosphate Degradation | 0.0288 | INPP5E,IMPA2 |
| Oxidative Ethanol Degradation III | 0.0288 | ALDH2,ALDH7A1 |
| Asparagine Degradation I | 0.0295 | ASPG |
| Proline Degradation | 0.0295 | LOC102724788/PRODH |
| Choline Degradation I | 0.0295 | ALDH7A1 |
| Sulfate Activation for Sulfonation | 0.0295 | PAPSS2 |
| Cysteine Biosynthesis/Homocysteine Degradation | 0.0295 | CBS/CBSL |
| Fatty Acid α-oxidation | 0.0324 | ALDH2,ALDH7A1 |
| Putrescine Degradation III | 0.0355 | ALDH2,ALDH7A1 |
| TR/RXR Activation | 0.0380 | PIK3C2G,APOA5,G6PC,SYT12 |
| Tight Junction Signaling | 0.0398 | NUDT21,PVRL3,VCL,CLDN7,NAPA,PRKAR1A |
| FAK Signaling | 0.0417 | CAPN8,PIK3C2G,VCL,EGFR |
| FXR/RXR Activation | 0.0417 | SLC27A5,APOA4,APOM,SERPINF1,G6PC |
| Tryptophan Degradation X (Mammalian, via Tryptamine) | 0.0417 | ALDH2,ALDH7A1 |
| Ethanol Degradation IV | 0.0417 | ALDH2,ALDH7A1 |
| UVA-Induced MAPK Signaling | 0.0427 | PLCE1,PIK3C2G,TNKS2,EGFR |
| 5-aminoimidazole Ribonucleotide Biosynthesis I | 0.0437 | PFAS |
| Thyroid Hormone Biosynthesis | 0.0437 | IYD |
| 4-aminobutyrate Degradation I | 0.0437 | SUCLG2 |
| Phototransduction Pathway | 0.0447 | GUCY1A3,OPN1LW,PRKAR1A |
| Superpathway of D-myo-inositol (1,4,5)-trisphosphate Metabolism | 0.0490 | INPP5E,IMPA2 |

^a^Pathway analysis was performed with Ingenuity Pathways Analysis ( IPA; Ingenuity Systems, Inc., Redwood City, CA, www.ingenuity.com) tool. Canonical pathways with significant p values (p value < 0.05) are listed.

^b^Enriched canonical pathways associated with the input gene list.

^c^P values calculated by Fisher's exact test right-tailed for gene enrichment analysis, It ranges from 0 to 1. Fisher's exact P Value = 0 represents perfect enrichment. P values smaller than 0.05 are considered strongly enriched in the canonical pathways.

^d^Molecules in the pathway overlapping with the input gene list.
